# The identification of mitochondrial DNA variants in glioblastoma multiforme

**DOI:** 10.1186/2051-5960-2-1

**Published:** 2014-01-02

**Authors:** Ka Yu Yeung, Adam Dickinson, Jacqueline F Donoghue, Galina Polekhina, Stefan J White, Dimitris K Grammatopoulos, Matthew McKenzie, Terrance G Johns, Justin C St John

**Affiliations:** 1 Mitochondrial Genetics Group, Centre for Genetic Diseases, Monash Institute of Medical Research, Monash University, 27-31 Wright Street, Clayton, Victoria 3168, Australia; 2 Division of Metabolic and Vascular Health, Warwick Medical School, University of Warwick, Gibbet Hill Road, Coventry CV4 7AL, UK; 3 The Oncogenic Signalling Laboratory, Centre for Cancer Research, Monash Institute of Medical Research, Monash University, 27-31 Wright Street, Clayton, Victoria 3168, Australia; 4 The Molecular Basis of Mitochondrial Disease Group, Centre for Genetic Diseases, Monash Institute of Medical Research, Monash University, 27-31 Wright Street, Clayton, Victoria 3168, Australia; 5 Centre for Cancer Research, Monash Institute of Medical Research, Monash University, 27-31 Wright Street, Clayton, Victoria 3168, Australia; 6 Biomedical Genomics Group, Centre for Genetic Diseases, Monash Institute of Medical Research, Monash University, 27-31 Wright Street, Clayton, Victoria 3168, Australia; 7 Pathology Services, University Hospital Coventry and Warwickshire NHS Trust, Clifford Bridge Road, Coventry CV2 2DX, UK

**Keywords:** Mitochondrial DNA, Tumorigenesis, Genetic variants, Aerobic glycolysis, Depletion, Oxidative phosphorylation

## Abstract

**Background:**

Mitochondrial DNA (mtDNA) encodes key proteins of the electron transfer chain (ETC), which produces ATP through oxidative phosphorylation (OXPHOS) and is essential for cells to perform specialised functions. Tumor-initiating cells use aerobic glycolysis, a combination of glycolysis and low levels of OXPHOS, to promote rapid cell proliferation and tumor growth. Glioblastoma multiforme (GBM) is an aggressively malignant brain tumor and mitochondria have been proposed to play a vital role in GBM tumorigenesis.

**Results:**

Using next generation sequencing and high resolution melt analysis, we identified a large number of mtDNA variants within coding and non-coding regions of GBM cell lines and predicted their disease-causing potential through *in silico* modeling. The frequency of variants was greatest in the D-loop and origin of light strand replication in non-coding regions. *ND6* was the most susceptible coding gene to mutation whilst *ND4* had the highest frequency of mutation. Both genes encode subunits of complex I of the ETC. These variants were not detected in unaffected brain samples and many have not been previously reported. Depletion of HSR-GBM1 cells to varying degrees of their mtDNA followed by transplantation into immunedeficient mice resulted in the repopulation of the same variants during tumorigenesis. Likewise, *de novo* variants identified in other GBM cell lines were also incorporated. Nevertheless, *ND4* and *ND6* were still the most affected genes. We confirmed the presence of these variants in high grade gliomas.

**Conclusions:**

These novel variants contribute to GBM by rendering the ETC. partially dysfunctional. This restricts metabolism to anaerobic glycolysis and promotes cell proliferation.

## Background

Mitochondrial DNA (mtDNA) resides exclusively within the matrix of the mitochondrion [1]. MtDNA is ~16.6 kb in size and encodes 13 proteins that are essential to the electron transfer chain, which produces the vast majority of cellular ATP through the biochemical process of oxidative phosphorylation (OXPHOS) [2]. It also consists of 22 tRNAs and 2 rRNAs and the D-Loop, which is the site of interaction for the nuclear-encoded factors that regulate mtDNA transcription and replication. The D-loop also contains two hypervariable regions, which are indicative of maternal ancestry [3].

The absence of intronic regions increases the likelihood of a variant residing within the coding regions of mtDNA [4], especially since the non-coding region accounts for only ~38% of the entire mitochondrial genome [5]. In the disease state, mutations are commonly observed throughout the mitochondrial genome [6,7]. Due to the presence of multiple copies of mtDNA in the matrix of each mitochondrion and cells having large numbers of mitochondria, mutant and wild-type mtDNA can co-exist.

Primarily, aerobic glycolysis is central to tumor cell metabolism, as these cells exhibit three-times higher glycolytic activity than their normal counterparts [8]. This reflects the Warburg effect, whereby cancer cells undergo a metabolic transition, regardless of the oxygen concentration available [9]. This causes the cell to favour glycolysis rather than OXPHOS, as glycolysis generates ATP at a higher rate than OXPHOS, which produces ATP at a higher yield [10], and is thus more effective for cellular proliferation and growth. It is therefore likely that the presence of multiple mtDNA variants in a single cell or cohort of cells would promote tumorigenesis as there would be a decreased ability to generate ATP through OXPHOS and an increased reliance on glycolysis.

Generally, there is an accumulation in the number of mtDNA variants before a cell experiences detrimental or phenotypic effects [11] and this is likely to be the case for tumor cells. Nevertheless, it remains to be determined whether mtDNA variants present in tumors arise from the clonal expansion of existing variants or if they occur *de novo*. Normally, variants must be present at significant levels to allow for their detection [12]. However, next generation sequencing enables whole mitochondrial genomes to be sequenced at a high coverage of over 1000-fold, which provides sufficient depth to identify sequence variants at even low levels [13,14].

Although the benefits of using such technology are clear, variants can be called that are interpreted as being true variants but are erroneous [15]. High resolution melt (HRM) analysis is an appropriate validation method to confirm next generation sequencing results by eliminating any errors that may have arisen from the sequencing reactions [16] or from long PCR amplification [17]. Confirmed variants can then be analysed by bioinformatics programs, such as SNPs & GO and MutPred that predict the impact of the SNPs on protein function [18].

Gliomas account for 80% of all primary malignant brain tumors and about 50% of gliomas give rise to Glioblastoma Multiforme (GBM) [19]. GBM has been rated most severe (level 4) on the World Health Organisation’s (WHO) scale of severity, due to its malignant nature [20]. For patients diagnosed with GBM, prognosis is poor with median survival estimated at 12–14 months [21]. Indeed, only about 3-5% of patients survive beyond 3 years [21].

A number of cell lines are representative of GBM. These include the frequently investigated lines, such as U87MG, SF-767 [22] and HSR-GBM1 [23]. Within patient GBM tissues, amplification of epidermal growth factor receptor (EGFR) genes is often observed [24]. This is evident in the GBM 6 and HK301 neurosphere cell lines, both of which express EGFRvIII. Others have been established from expanded cultures from high-grade human GBM tumors.

We have analysed a series of GBM cell lines using a combination of next generation sequencing and HRM analysis to identify variants within the mitochondrial genome that are indicative of tumorigenesis in GBM. We have then depleted one of the cell lines to varying degrees to determine whether the same variants reoccur following mtDNA replenishment as tumorigenesis progresses. We have applied several bioinformatic approaches to predict the severity of each mutation and to model the effects on protein structure. We have confirmed that these variants are indicative of GBM through their identification in GBM tumors. The frequency of variants is greatest within the D-loop and the origin of light strand replication in the non-coding regions whilst NADH dehydrogenase 6 (*ND6*) is the most susceptible gene for the development of mutations in the coding region.

## Methods

### Cell culture

The GBM cell lines GBM L1, GBM L2, GBM4, GBM6, CSC020, CSC014, BAH-1 and HK301 were established in-house directly from patient tumors using neural stem cell conditions, as recently described by us [25]. None of these cell lines was exposed to fetal calf serum (FCS). HSR-GBM1, GBM L1, GBM L2 and hNSCs derived from the NIH-approved H9 (WA09) human ESC line (Invitrogen, Carlsbad, CA, USA) were cultured as neurospheres on ultra-low attachment plates (Sigma, St. Louis, MO) in DMEM/F12 Media containing 2% StemPro neural supplement (both Gibco, Carlsbad, CA, USA), 20 ng/ml basic fibroblast growth factor (bFGF) and 20 ng/ml epidermal growth factor (EGF; both Millipore, Billerica, MA). They were passaged every 3 days to maintain their undifferentiated states. The GBM4, GBM6, CSC020, CSC014, BAH-1 and HK301 cell lines were cultured in the same manner but with knockout DMEM/F12 media and with the addition of GlutaMAX (2 mM) only for the GBM 4, CSC-020 and CSC-014 cell lines. The NO7-152 cell line is an in-house cell line and is an early passage line grown in the traditional manner using DMEM high glucose, 4% non-essential amino acids (NEAA), 20% FCS and 2 mM GlutaMAX (all from Gibco, Carlsbad, CA, USA). Both U87MG (sourced directly from ATCC) and SF-767 (sourced directly from Brain Tumor Research Center Tissue Core, UCSF) adherent cell lines were cultured in DMEM/F12 Media supplemented with 5% FCS. Cells underwent enzymatic passaging every 3 days using TrypLE™ Express (Gibco) to dissociate cells into a single cell suspension.

### MtDNA depletion

HSR-GBM1 cells were cultured as described above but in the presence of the mtDNA depletion agent, 2′3′-dideoxycytidine (ddC), which was added daily during half media changes at a concentration of 10 μg/ml, alongside 50 μg/ml of uridine (both: Sigma, St Louis, MO). Cells were passaged every 3 days. They were allowed to recover in vitro by withdrawal of ddC from the culture media.

### Normal brain and tumor tissue samples

Normal human brain and tumor tissue samples were obtained from the Victorian Brain Bank Network under the human research ethics number 09023B, Southern Health, HREC. The normal human tissues were obtained from individuals with no known history of neoplastic or neurological pathology. The brain tumor samples were from patients diagnosed with high grade glioma.

### Xenograft tumor models

Mouse experiments were approved by the Animal Ethics Committee, Monash University, Approval Number: MMCA/2011/76. HSR-GBM1 cells were depleted of their mtDNA over 50 days in culture. They were harvested after depletion to 50%, 20%, 3% and 0.2% of their original mtDNA content. Following depletion, 5 × 10^5^ cells were resuspended in 100μl of Complete DMEM/StemPro Media and inoculated subcutaneously into both leg regions of immunedeficient female BALB/c nude mice (Animal Research Centre, Perth, Australia) aged between 4 to 6 weeks old. Tumors were extracted and stored at -80°C.

### DNA extraction

DNA was extracted from cell pellets and tumors using the DNeasy Blood & Tissue kit in the presence of RNase A (Qiagen, West Sussex, UK), following the manufacturer’s recommendations.

### Long PCR

Templates for next generation sequencing were generated by long PCR amplification of two overlapping fragments that each spans half of the mitochondrial genome. 50 ng DNA template was used per reaction, together with 1× High Fidelity PCR buffer, 100 mM MgSO_4_, 1 mM dNTPs (Bioline, London, UK), 1U of Platinum Taq High Fidelity (Invitrogen, Carlsbad, CA, USA) in the presence of 10 μM each of the forward and reverse primers (long1 F: 5′-GACGGGCTCACATCACCCCATAA-3′; long1 R: 5′-GCGTACGGCCAGGGCTATTGGT-3′; long2 F: 5′-GCCACAACTAACCTCCTCGGACTCCT-3′; long2 R: 5′-GGTGGCTGGCACGAAATTGACC-3′). All reactions were performed in a total volume of 50μl. Conditions were 94°C for 2 minutes, 35 cycles of 94°C for 15 seconds, 63°C for 30 seconds and 68°C for 8 minutes 45 seconds. Products were purified using the QIAquick PCR purification kit (Qiagen).

### Next generation sequencing using the Ion Torrent Personal Genome Machine (PGM)™

Purified amplicon pairs generated from long PCR were combined at equal concentrations, prior to generation of the amplicon libraries. Amplicon libraries were generated using the recommended workflow procedures from the Ion Fragment Library Kit and Ion Xpress™ Template kit (Life Technologies). MtDNA was sheared using the Covaris Adaptive Focused Acoustics™ system. Fragments of approximately 200 bp were selected following electrophoretic separation with the E-gel system (Life Technologies). Confirmation of product and the quality of the mtDNA was assessed by the Agilent Bioanalyzer using the Agilent High Sensitivity DNA Kit (Agilent, Santa Clara, CA). For multiplexing of the samples, each DNA library was barcoded using different ligation adaptors. Libraries were then pooled at equal concentrations and loaded onto 316 chips for sequencing. Sequence alignment to the reference genome was performed using the Ion Torrent Suite (v.2.2).

Variant selection was performed using CLC Genomics Workbench (v5.5.1). For quality control, reads were filtered to exclude those of a nucleotide length of <15 bp, with one nucleotide being trimmed from both ends of each read. All reads accepted into analysis surpassed a Phred quality score of 15. The following parameters were applied to score reads during the selection process for inclusion into the final alignment: setting a mismatch cost of 2 and an insertion/deletion cost of 3; acceptance of reads that had a minimum of 80% identity to the reference sequence; and exclusion of all duplicate reads. For single nucleotide polymorphism (SNP) analysis, a minimum mutation threshold of 3% was applied.

### HRM analysis

Genomic DNA samples for PCR were loaded on 96-well plates (Bio-Rad Laboratories, Hercules, CA) with each sample analysed in triplicate. The starting template for all reactions was 10 ng in total, amplified in the presence of 1× HRM master mix containing LCGreen® Plus + (TrendBio), and 2.5 μM of forward and reverse primers (Additional file 1: Table S1). Reactions were performed in a total volume of 10 μl, with an overlay of 20 μl mineral oil (Sigma). Amplification conditions were: 95°C for 2 minutes, 45 cycles of 94°C for 30 seconds and 55°C for 30 seconds, followed by 1 cycle at 94°C for 30 seconds and cooling to 25°C for heteroduplex formation.

Products underwent melt analysis on the LightScanner (Idaho Technologies, Salt Lake City, Utah) with analysis performed using the LightScanner Instrument & Analysis software with Call-IT 2.0 (V.2.0.0.1331). Data acquisition began at 70°C and increased incrementally by 0.1°C until the reaction terminated at 96°C [26].

Validation of next generation sequencing by HRM is described in: Additional file 2: Supplementary Methods.

### In silico predictive analysis on protein function

Web application tools SNPs & GO (University of Bologna) and MutPred (v.1.2.) (Buck Institute, Indiana University) were used to predict the potential effects of amino acid changes to protein function. SNPs & GO uses the known protein sequence, protein function and evolutionary data based on the Swiss-Prot protein database to determine whether the variant is ‘neutral’ or capable of causing ‘disease’, and provides a reliability value in association with its prediction [27]. MutPred predicts the top five features within the protein that are likely to occur as a result of the amino acid substitution, and provides a probability value for each prediction given [28].

### Molecular protein modeling

Protein sequences were obtained from the National Center for Biotechnology Institute database. The Clustal Omega tool (EMBL-EBI) was used for sequence alignment. If a protein structure shared a sequence identity of over 70% to a protein of interest, the location of the mutation was identified and its effect predicted using Swiss-PDB viewer (v.4.1.0). Protein structures were obtained from the RCSB Protein Data Bank (PDB) database (PDB ID for COX I: 1OCC_A, COX II: 1OCC_B, COX III: 1V54_C, CYT B: 1BGY_C, CYT C1: 1BGY_D, Rieske protein: 1BGY_E).

## Results

### Validation of the Ion Torrent PGM™ to detect a range of sequence variants

PCR products containing a known mtDNA variant and its wild-type counterpart were sub-cloned into DNA plasmids. They were mixed at varying percentages to generate a series of standard dilutions to test the sensitivity of the Ion Torrent and were validated by HRM (Additional file 1: Table S1; Additional file 3: Figure S1A-C).

### Identification of variants in the GBM cell lines

The full mitochondrial genomes of 12 GBM cell lines were then analysed by Ion Torrent to determine whether the pattern of variants present are shared amongst all cell lines. Altogether, 13 variants were detected in the non-coding region (Table 1) and 19 in the coding region of the mitochondrial genome (Table 2). Within the non-coding region, the majority of variants occurred within the triple-stranded D-loop region. The D-loop harbored the highest frequency of mutation (77.9%) at position 16224 bp in the U87MG cells. For GBM L1, GBM L2 and NO7-152 cell lines, the occurrence of a variant at 310 bp of the D-loop region was shared between the three lines at similar frequencies whilst it was three-fold higher in the CSC014 cells. Variants at positions 2130 bp and 5752 bp were also common, with 6 lines sharing the presence of both variants at similar levels (GBM L1, GBM L2, GBM 4, CSC014, CSC020 and NO7-152). Likewise, the GBM 6, SF-767, U87MG, HK301 and BAH1 lines shared the presence of the 16519 bp variant at high levels (range 51.2 to 58.2%), whilst the GBM 6 and U87MG cell lines shared the presence of the 3168 bp variant (*16 s rRNA*) at a frequency of 3.7%. For some shared variants, the frequency differed considerably between lines. For example, the D-loop variant at 302 bp was present in the GBM L1 cells at 16.4%, approximately two-fold lower than for NO7-152 (at 39.9%) and CSC014 (at 41.5%) cells.

**Table 1:** MtDNA variants identified from sequencing the non-coding region of mtDNA using the Ion Torrent PGM on 12 cell lines representative of GBM

			**Percentage change in variant (%)**												
**Reference position**	**Reference**	**Variant**	**HSR-GBM1**	**GBM L1**	**GBM L2**	**GBM 4**	**GBM 6**	**CSC 014**	**CSC 020**	**NO7 152**	**SF-767**	**U87MG**	**HK301**	**BAH1**	**Gene region**
16186	C	C/T	4.5												D loop - Hypervariable segment 1, 7S DNA, membrane attachment site
16218	C	C/T	16.9												
16224	T	T/C										77.9			
16519	T	T/C					53.7				55.8	58.2	51.2	52.9	D loop - 7S DNA, membrane attachment site
194	C	T/C				4.1									D loop - Hypervariable segment 2, membrane attachment site
302	A	A/C		16.4				41.5		39.9					
310	T	T/C		5.6	5.0			17.5		6.0					
1386	T	T/C	9.9												12s rRNA
**2002**	G	G/A					11.1								16s rRNA
**2130**	A	A/G		3.9	5.5	4.0		3.6	6.3	7.4					
**2817**	G	G/A												11.7	
**3168**	C	C/T					3.7					3.7			
5752	A	A/G		11.3	13.1	11.4		10.5	10.9	9.9					L strand replication origin

Variants represent those that arise at a percentage frequency of or greater than 3%, after strict parameters were applied to exclude low quality reads from the final mapping of each full mitochondrial genome sequence. Variants that have not been previously reported are in bold.

For the 19 variants identified within the coding region of the mitochondrial genome (Table 2), the levels ranged from 3.1% at position 12101 bp in the NADH dehydrogenase 4 (*ND4)* gene in the CSC-014 cell line to 55.5% at position 8252 bp (Cytochrome C Oxidase II; *COX II*) in the GBM L1 cell line. The most frequently observed variants were at positions 11512 bp (*ND4*) and 14160 bp (*ND6*), which were present in 5/12 and 8/12 lines, respectively. 16 of the variants resulted in non-synonymous amino acid changes whilst 3 were synonymous. Of the 16 non-synonymous substitutions, 5 were predicted by the *in silico* web-based tool, SNPs & GO, to be disease causing (Table 2). The 13061 bp variant was predicted by MutPred to cause impaired protein function due to the substitution of a glutamine for a proline at position 242 in the NADH dehydrogenase 5 (ND5) polypeptide chain (P = 0.035) resulting in the loss of glycosylation. The variant at position 14159 bp substituted a proline for a methylated arginine at position 172, again disrupting protein function (P = 0.03). The cytochrome B (*CYT B*) disease-causing variants at positions 15264 bp and 15267 bp were predicted to result in the loss of relative solvent accessibility and gain of glycosylation, respectively.

**Table 2:** MtDNA variants identified within the coding region of 12 GBM cell lines

		**Percentage change in variant (%)**															**MutPred**	
**Reference position**	**Variant change**	**HSR-GBM1**	**GBM L1**	**GBM L2**	**GBM 4**	**GBM 6**	**CSC 014**	**CSC 020**	**NO7 152**	**SF-767**	**U87MG**	**HK301**	**BAH1**	**Gene region**	**Amino acid change**	**SNPs & GO**	**Probability of deleterious mutation**	**Top 5 predicted features caused by the amino acid mutation**
6422	C→T	3.8												COX I	Syn (P)	-	-	
**6999**	G→A	6.5													V366M	Neutral, RI 4, uniprot P00395	0.454	Loss of stability (P = 0.0688)
																		Loss of sheet (P = 0.0817)
																		Loss of catalytic residue at V366 (P = 0.1011)
																		Loss of glycosylation at S362 (P = 0.2022)
																		Gain of loop (P = 0.4661)
8251	G→A		43.5		50.8									COX II	Syn (G)	-	-	
**8252**	C→A		55.5		46.8										P223T	Neutral, RI 9, uniprot P00403	0.327	Gain of glycosylation at P223 (P = 0.1135)
																		Loss of disorder (P = 0.1694)
																		Loss of catalytic residue at G222 (P = 0.2169)
																		Loss of phosphorylation at T226 (P = 0.3735)
																		Loss of helix (P = 0.3949)
**10473**	C→G						4.3							ND4L	P2A	Neutral, RI 9, uniprot P03901	0.344	**Loss of disorder (P = 0.0496)**
																		Loss of catalytic residue at L3 (P = 0.1395)
																		Gain of helix (P = 0.2684)
																		Loss of loop (P = 0.3664)
																		Loss of phosphorylation at Y5 (P = 0.4053)
**10814**	A→C		6.0		5.3			5.0	5.2					ND4	K19Q	Neutral, RI 8, uniprot P03905	0.551	**Loss of methylation at K19 (P = 0.0012)**
																		**Loss of ubiquitination at K19 (P = 0.0283)**
																		Loss of MoRF binding (P = 0.134)
																		Gain of helix (P = 0.2684)
																		Loss of catalytic residue at K19 (P = 0.2966)
11361	T→C						6.0								M201T	Neutral, RI 5, uniprot P03905	0.706	Loss of stability (P = 0.0853)
																		Gain of ubiquitination at K206 (P = 0.1204)
																		Gain of catalytic residue at M201 (P = 0.1253)
																		Gain of methylation at K206 (P = 0.1903)
																		Loss of MoRF binding (P = 0.2081)
11512	C→A		6.0		9.6		11.0	6.4	15.2						N251K	Neutral, RI 4, uniprot P03905	0.495	**Gain of methylation at N251 (P = 0.0194)**
																		Gain of MoRF binding (P = 0.0632)
																		Loss of stability (P = 0.0709)
																		Loss of ubiquitination at K255 (P = 0.0768)
																		Gain of solvent accessibility (P = 0.0837)
11674	C→T				3.5										Syn (T)	-	-	
**12101**	T→C				3.8		3.1		4.2						S448P	Neutral, RI 7, uniprot P03905	0.484	Loss of helix (P = 0.0093)
																		Gain of loop (P = 0.0321)
																		Gain of relative solvent accessibility (P = 0.09)
																		Gain of sheet (P = 0.1451)
																		Gain of catalytic residue at L447 (P = 0.1502)
12102	C→T				3.7				3.2						S448F	Neutral, RI 3, uniprot P03905	0.472	Loss of disorder (P = 0.0619)
																		Gain of helix (P = 0.2059)
																		Loss of loop (P = 0.2897)
																		Loss of phosphorylation at S448 (P = 0.5302)
																		Gain of catalytic residue at S448 (P = 0.5425)
**12877**	G→C									27.7				ND5	G181R	Disease, RI 7, Uniprot P03915	0.795	Loss of catalytic residue at I183 (P = 0.1945)
																		Gain of MoRF binding (P = 0.2553)
																		Gain of methylation at G181 (P = 0.3559)
																		Loss of helix (P = 0.4763)
																		Loss of stability (P = 0.5598)
13043	C→T										3.3				A236V	Neutral, RI 3, Uniprot P03915	0.786	Loss of glycosylation at P234 (P = 0.0757)
																		Loss of disorder (P = 0.0789)
																		Gain of helix (P = 0.132)
																		Loss of phosphorylation at T241 (P = 0.2504)
																		Loss of loop (P = 0.2897)
**13061**	C→A							4.0							P242Q	Disease, RI 0, uniprot P03915	0.776	**Loss of glycosylation at P242 (P = 0.035)**
																		Loss of phosphorylation at T241 (P = 0.1079)
																		Loss of disorder (P = 0.1807)
																		Loss of catalytic residue at E238 (P = 0.1978)
																		Loss of helix (P = 0.2271)
**14159**	C→G			4.1										ND6	R172P	Disease, RI 3, uniprot P03923	0.423	**Loss of methylation at R172 (P = 0.0305)**
																		Gain of catalytic residue at R172 (P = 0.0632)
																		Loss of sheet (P = 0.0817)
																		Loss of stability (P = 0.126)
																		Gain of disorder (P = 0.1619)
**14160**	G→C			4.0	3.5	3.3	7.5		4.3	3.2		8.1	5.0		R172G	Neutral, RI 2, uniprot P03923	0.442	**Loss of methylation at R172 (P = 0.0305)**
																		Loss of stability (P = 0.0532)
																		Loss of sheet (P = 0.0817)
																		Gain of disorder (P = 0.1578)
																		Gain of loop (P = 0.2045)
**14426**	C→T						8.9								G85E	Neutral, RI 7, uniprot P03923	0.364	**Loss of glycosylation at S84 (P = 0.0357)**
																		**Gain of solvent accessibility (P = 0.0456)**
																		Loss of catalytic residue at V86 (P = 0.1017)
																		Gain of disorder (P = 0.1294)
																		Gain of loop (P = 0.2045)
**15264**	C→T	14.1												CYTB	P173L	Disease, RI 4, uniprot P00156	0.361	Loss of relative solvent accessibility (P = 0.0793)
																		Loss of solvent accessibility (P = 0.089)
																		Gain of methylation at R177 (P = 0.1226)
																		Loss of glycosylation at S172 (P = 0.1763)
																		Loss of disorder (P = 0.2084)
**15267**	C→G	20.5													T174S	Disease, RI 3, uniprot P00156	0.866	Gain of glycosylation at T174 (P = 0.0587)
																		Gain of disorder (P = 0.0665)
																		Loss of catalytic residue at T174 (P = 0.1513)
																		Loss of methylation at R177 (P = 0.2045)
																		Loss of sheet (P = 0.3635)

*In silico* analysis was performed using the online tools SNPs & GO and MutPred to predict the impact these variants exert on the corresponding protein of interest. Variants that have not been previously reported are in bold.

Overall, the majority of variants in the coding regions for all 12 cell lines arose in the *ND4* region (n = 6 variants). Both the *ND5* and *ND6* genes, the latter of which is encoded on the light strand of mtDNA, possessed the second highest number of variants (n = 3). The *ND6* variant at position 14160 bp was the most common mutational event with 8/12 lines harboring this variant. All variants were confirmed using HRM analysis, for example see Additional file 4: Figure S2.

### The susceptibility of the different mtDNA gene regions to the development of variants

To determine the susceptibility of various mtDNA regions to mutation, we normalized the number of variants identified in each mtDNA region to the size of the gene region (bp) on which the variant was located. The overall probability of deriving a mutation in the non-coding region (2.08 × 10^-3^) was greater than in the coding region (1.84 × 10^-3^) (Additional file 5: Table S2). The *12S rRNA* region was found to be the least susceptible to mutation of all the non-coding regions (n = 1 variant within 953 bp: Additional file 5: Table S2) whilst the origin of light strand replication (1 variant within 77 bp) was the region most likely to develop mutation (12.38X greater than for the *12S rRNA* region) (Additional file 5: Table S3). Although the D-loop harboured 7 variants, due to its size, it was the second most susceptible non-coding region to mutation. In the coding region, Cytochrome C Oxidase I *(COX I)* was the least susceptible to acquiring mutation (1.298 × 10^-3^), whilst *ND6* was the most susceptible (5.725 × 10^-3^), representing a 4.41X fold difference between the two genes (Additional file 5: Table S3).

### Protein modeling of non-synonymous amino acid substitutions

To understand the impact of non-synonymous amino acid changes induced by the introduction of variants into the mitochondrial genome, we performed protein modeling on the bovine protein structures for COX I, COX II and CYT B equivalents due to the unavailability of the human structures. These are 91%, 72% and 78.4% homologous to their respective human proteins. The ND subunits do not have protein structures available that closely resemble the corresponding human protein sequence.

**Figure 1:**
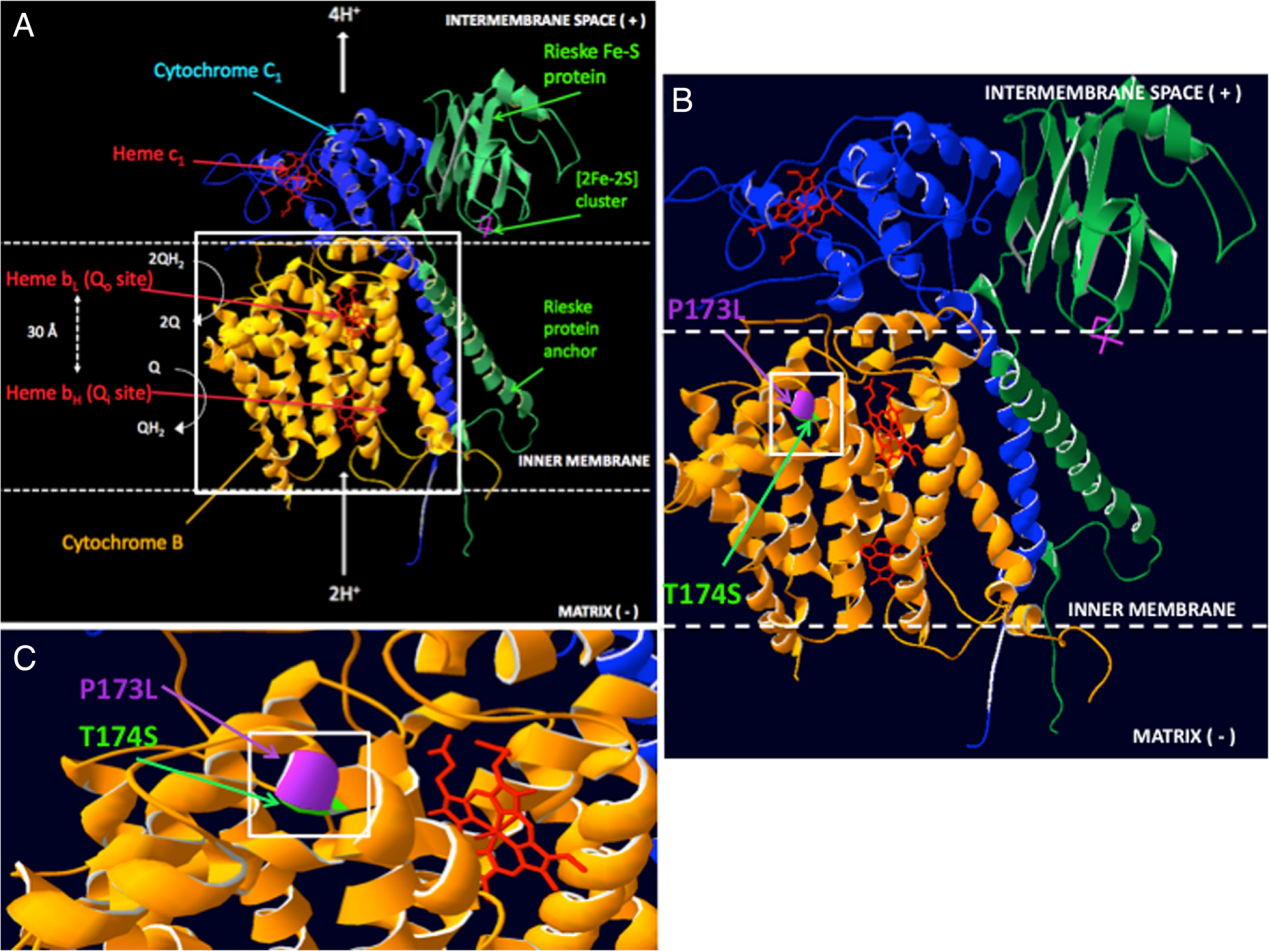
**Protein structure of the complex III catalytic centre (the cytochrome bc1 complex) with annotated locations for the variants in cytochrome B. (A)** The catalytic centre of complex III is shown, represented by three interacting subunits, cytochrome B (yellow), cytochrome C_1_ (blue) and the Rieske iron-sulfur protein (green). The key redox cofactors are Heme b_L_ and b_H_, which reside within cytochrome B, and heme c_1_ in cytochrome C_1_. Oxidation of ubihydroquinone (QH_2_) occurs at the Q_o_ site, and reduction of ubiquinone to ubiquinol at the Q_i_ site. Following these events, a membrane potential is generated across the inner mitochondrial membrane. The Rieske iron-sulfur protein interconnects the cytochrome B and C_1_ subunits. **(B)** Location of the proline to leucine (P173L) and threonine to serine (T174S) mutations are highlighted on cytochrome B. Both of these reside on a transmembrane helical region of the subunit. **(C)** Magnified view of the adjacent P173L and T174S mutations.

The CYT B variants at mtDNA nucleotide positions 15264 bp and 15267 bp induce a transition from proline to leucine at position 173 (P173L) for the variant at 15264 bp, and a change from threonine to serine at position 174 (T174S) for the 15267 bp variant. These variants are located at the terminal end of one of the transmembrane helical domains on CYT B (Figure 1). However, their relatively central location in relation to the catalytic centre of CYT B is predicted to impact on function. The mtDNA variant identified at position 6999 bp in the COX I region of the HSR-GBM1 cells induces a change from valine to methionine at position 366 of the polypeptide chain. However, the mutational change resides within a transmembrane helix region that faces towards the mitochondrial intermembrane space (Additional file 6: Figure S3) and is predicted to have a neutral effect (Table 2). The variant at position 8252 bp in COX II of the HSR-GBM1 cells results in a proline to a threonine transition at position 223 (P223T) of the protein sequence. It maps to a helix region located away from the catalytic core of complex IV in the mitochondrial intermembrane space (Additional file 7: Figure S4) and is thus predicted to be neutral (Table 2).

### Variants identified in the brain tissue of normal brain biopsy patients

In order to determine the type and frequency of mtDNA variants in brain samples from unaffected individuals, we analysed brain tissue samples from 13 individuals with no known brain disease. The majority of variants (14/21) were present in hypervariable regions I and II of the D-loop (Table 3). Sample 05–470 possessed the highest number of non-coding variants (n = 12) whilst the remaining 12 samples possessed 5 or fewer. Apart from the variant at position 16519 bp, the other variants were present at frequencies <10% except for the A→G substitution at position 16293 bp, which was present at 16.1% in 04–273, and the G→C substitution at 12.1% at position 2647 bp in the *16 s rRNA* region for sample 05–470.

**Table 3:** Variants detected within the non-coding region of mtDNA in normal brain tissue (n = 13)

			**Percentage change in variant (%)**													
**Reference position**	**Reference**	**Variant**	**03-965**	**04-273**	**05-470**	**07-104**	**07-445**	**07-635**	**08-012**	**08-026**	**09-005**	**09-152**	**16-1**	**22-1**	**20-1**	**Gene region**
16069	C	T			7.2											D loop - Membrane attachment site, Hypervariable segment 1
16126	T	C			5.2											D loop - Membrane attachment site, Hypervariable segment 1, 7S DNA
16145	G	A			4.8											D loop - Membrane attachment site, Hypervariable segment 1, 7S DNA, termination-associated sequence
16179	C	T									4.3					D loop - Membrane attachment site, Hypervariable segment 1, 7S DNA
16222	C	T			5.1											
16261	C	T			9.4											
16293	A	G		16.1												
16356	T	C									3.0					
16519	T	C	57.5		54.5			56.0		57.6	58.3	59.4				D loop - Membrane attachment site, 7S DNA
9	G	T			5.8											
10	T	C			5.8											
66	G	T													3.2	D-loop - Hypervariable segment 2, membrane attachment site, 7S DNA
73	A	G			6.2											
150	C	T									4.4					D loop - Hypervariable segment 2, H-strand origin, Membrane attachment site, 7S DNA
189	A	G							3.0							
195	T	C									4.5					D loop - Hypervariable segment 2, H-strand origin, Membrane attachment site
242	C	T			7.7											D loop - Hypervariable segment 2, H-strand origin, mtTF1 binding site, Membrane attachment site
751	A	T							3.9							12s ribosomal RNA (rRNA)
**2232**	A	T						3.0								16s ribosomal RNA (rRNA)
**2647**	G	C			12.1											
3010	G	A			3.5											

Variants represented those that were present at or above the 3% mutation threshold selection criteria. Variants that have not been previously reported are in bold.

In the coding region of the normal samples, 39 variants were identified (Table 4). *CYT B* harboured the largest proportion of variants (9/39) whilst *ND4* possessed the second highest proportion of variants (6/39). The majority of variants (21/39) led to non-synonymous amino acid changes, whilst 1 variant generated a stop codon at position 8630 bp.

**Table 4:** Variants identified in the coding region of the mitochondrial genome in normal brain tissue samples

			**Percentage change in variant (%)**																**MutPred**	
**Reference position**	**Reference**	**Variant**	**03-965**	**04-273**	**05-470**	**07-104**	**07-445**	**07-635**	**08-012**	**08-026**	**09-005**	**09-152**	**16-1**	**22-1**	**20-1**	**Gene region**	**Amino acid change**	**SNPs & GO**	**Probability of deleterious mutation**	**Top 5 predicted features caused by amino acid mutation**
4646	T	C									3.4					ND2	Syn (Y)	-		
**4701**	A	T				4.8											N78Y	Neutral, RI 7, Uniprot P03891	0.329	
4703	T	C				4.8											Syn (N)	-		
**4878**	G	A									4.6						A137T	Neutral, RI 5	0.786	
**4879**	C	G									5.0						A137G	Neutral, RI 5	0.78	**Loss of stability (P = 0.0465)**
6146	A	G									3.1					COX I	Syn (W)	-		
**8497**	A	G		12.5							4.8					ATP8	Syn (M)	-		
**8630**	A	G											3.7			ATP6	K35 Stop	Uniprot P00846		
8756	T	C								6.5							I77T	Neutral, RI 5	0.684	**Loss of stability (P = 0.0062)**
8790	G	A					18.4										Syn (L)	-		
8994	G	A								4.7							Syn (L)	-		
9070	T	G									6.2						S182A	Neutral RI 5	0.162	**Loss of glycosylation at S182 (P = 0.0094)**
9258	C	T					3.2									COX III	Syn (L)	-		
**9526**	C	G		5.0				3.8									A107G	Neutral, RI 5, Uniprot P00414	0.667	**Loss of stability (P = 0.0332)**
**9528**	C	A		9.9	6.4				4.8								P108T	Neutral, RI 2	0.731	
**9558**	C	A					5.8			3.6							P118T	Disease, RI 3	0.579	**Loss of catalytic residue at P117 (P = 0.0195)**
																				**Gain of glycosylation at P118 (P = 0.0283)**
10398	A	G			5.4											ND3	T114A	Neutral, RI 10, Uniprot P03897	0.071	
10993	G	A			4.6											ND4	Syn (M)	-		
11332	C	T									3.4						Syn (A)	-		
11467	A	G									3.1						Syn (L)	-		
**11516**	C	A							4.9								L253M	Neutral, RI 7, Uniprot P03905	0.369	
**11725**	A	T							3.0								Syn (T)	-		
**11791**	C	T											11.8				Syn (L)			
**12719**	T	C	3.2													ND5	M128T	Disease, RI 2, Uniprot P03915	0.795	**Loss of stability (P = 0.0126)**
12774	C	T											9.3				Syn (G)			
13984	C	T										3.7					Syn (L)	-		
**13985**	T	C			3.1						3.1	3.7					L550P	Neutral, RI 1	0.457	**Gain of loop (P = 0.0013)**
																				**Loss of helix (P = 0.0041)**
																				**Gain of catalytic residue at P549 (P = 0.0244)**
																				**Gain of glycosylation at L550 (P = 0.0364)**
																				**Gain of relative solvent accessibility (P = 0.0479)**
14155	C	T										3.8				ND6	Syn (G)	-		
**14159**	C	G							12.5								R172P	Disease, RI 3, Uniprot P03923	0.423	**Loss of methylation at R172 (P = 0.0305)**
**14160**	G	C					8.1		12.5								R172G	Neutral, RI 2	0.442	**Loss of methylation at R172 (P = 0.0305)**
14770	C	A												8.0		CYT B	N8K	Neutral RI 1, Uniprot P00156	0.446	**Gain of methylation at N8 (P = 0.0039)**
																				**Gain of MoRF binding (P = 0.0159)**
																				**Gain of ubiquitination at N8 (P = 0.0452)**
**14823**	A	C					4.0										N26T	Disease, RI 6	0.752	
14857	T	C							3.8								Syn (L)	-		
**14861**	G	T							3.6								A39S	Neutral, RI 5	0.33	
14866	C	T									5.4						Syn (C )	-		
15287	T	C					26.9										F181L	Neutral, RI 3	0.617	
15452	C	A			3.2												L236I	Neutral, RI 7	0.307	
**15579**	A	T						3.7									Y278F	Disease, RI 6	0.796	
15693	T	C									4.9						M316T	Neutral, RI 8	0.312	

Variants represented those that were present at or above the 3% mutation threshold selection criteria. Variants that have not been previously reported are in bold. Predictions on the effects of amino acid substitutions were performed using MutPred and SNPs & GO online tools.

The D-loop region was the most susceptible to mutation (1.517 × 10^-2^) amongst the coding and non-coding regions of mtDNA with *COX I* being the least susceptible (6.4893 × 10^-4^) (Additional file 5: Table S4) representing a 23.37-fold difference (Additional file 5: Table S5). Within the coding region, *CYT B* was the most susceptible amongst all other coding genes with a score 12.17X higher than that for *COX I* (Additional file 5: Table S5).

### Identification of variants through cell culture

In order to determine whether the culture environment would induce mtDNA variants over time, we sequenced the mitochondrial genome of a human neural stem cell (hNSC) line that had been subjected to multiple passaging (x20). We identified 2 variants in the non-coding region, which were restricted to the D-loop region. The variant at position 16519 bp had the highest percentage change at 51.3% whilst the variant at position 496 was present at 3.3%. In the coding region, there were 4 variants, namely three located in *ND5* at positions 13762 bp (3.2%), 13984 (3.6%) (D-loop) and 13985 (3.6%) and 1 residing in *CYT B* at position 15153 bp (9.4%). The low level of percentage variant present reflects the low levels identified in the normal samples. Furthermore, comparison of the variants present in the traditional immortalised lines (NO7 152, SF-767 and U87MG) and the primary glioma lines (GBM L1; GBM L2; GBM 4; GBM 6; CSC014; CSC020, HK301 and BAH1) did not reveal a greater distribution of mtDNA variants for one set of lines.

### Phylogenetic analysis of the GBM cell lines and normal brain samples

In order to determine that the normal samples and the GBM cell lines were not indicative of tight cohorts of mtDNA clusters but were from comparable lineages, we subjected them to phylogenetic analysis. As can be seen from Additional file 8: Figure S5, there is no bias due to the equal distribution of samples across a wide mtDNA genetic distribution and that the variants observed appear to be unique to the tumor samples.

### Identification of mtDNA variants essential to tumorigenesis

In order to determine which mtDNA variants identified in the GBM cell lines are essential to tumorigenesis, we depleted HSR-GBM1 cells to varying degrees, namely to 50% (mtDNA^50^), 20% (mtDNA^20^), 3% (mtDNA^3^) and 0.2% (mtDNA^0.2^) of their original mtDNA content. We transplanted these and non-depleted HSR-GBM1 (mtDNA^100^) cells into BALB/c nude mice and assessed whether the same or other mtDNA variants were selected for as cells replenished their mtDNA content during tumorigenesis.

**Figure 2:**
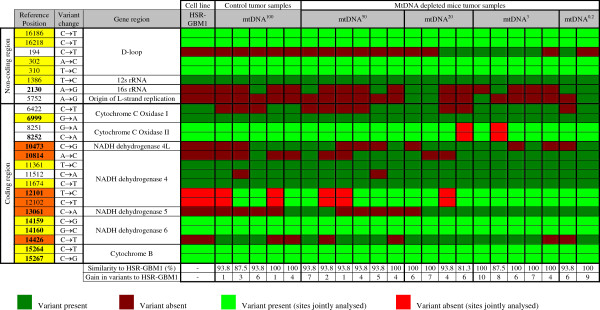
**HRM analysis of HSR-GBM1 cells that have undergone mtDNA depletion for up to 50 days, followed by the replenishment of mtDNA *in vivo *in immunedeficient nude mice, based on the panel of variants identified across the different GBM cell lines.** Tumors mtDNA^50^, mtDNA^20^, mtDNA^3^ and mtDNA^0.2^ were generated with HSR-GBM1 cells depleted of their mtDNA copy number to 50%, 20%, 3% and 0.2% of their original copy number relative to non-depleted HSR-GBM1 cells (mtDNA^100^). Multiple samples for each tumor type represent experimental replicates. The variants highlighted in yellow indicate variants shared between all samples analysed. Variants highlighted in orange represent those that have been acquired *de novo* during tumor formation and were identified within the other GBM cell lines.

Through HRM, we determined that 10/22 of the tumors harboured all of the variants present in the parental cells whilst 9 (2 mtDNA^100^, 5 mtDNA^50^, 1 mtDNA^20^ and 1 mtDNA^0.2^) shared 15/16 variants. Another 2 tumors (1 mtDNA^100^ and 1 mtDNA^3^) shared 14/16 variants and 1 (mtDNA^20^) shared 13/16 variants (Figure 2). In the non-coding region, the variants at 16186 bp, 16218 bp, 302 bp, 310 bp and 1386 bp (12 s rRNA) persisted in all tumors, whereas the variants at 6999 bp (COX I), 11361 bp and 11674 bp (ND4), 14159 bp and 14160 bp (ND6), and 15264 bp and 15267 bp (CYT B) persisted in the coding region of all tumors.

**Figure 3:**
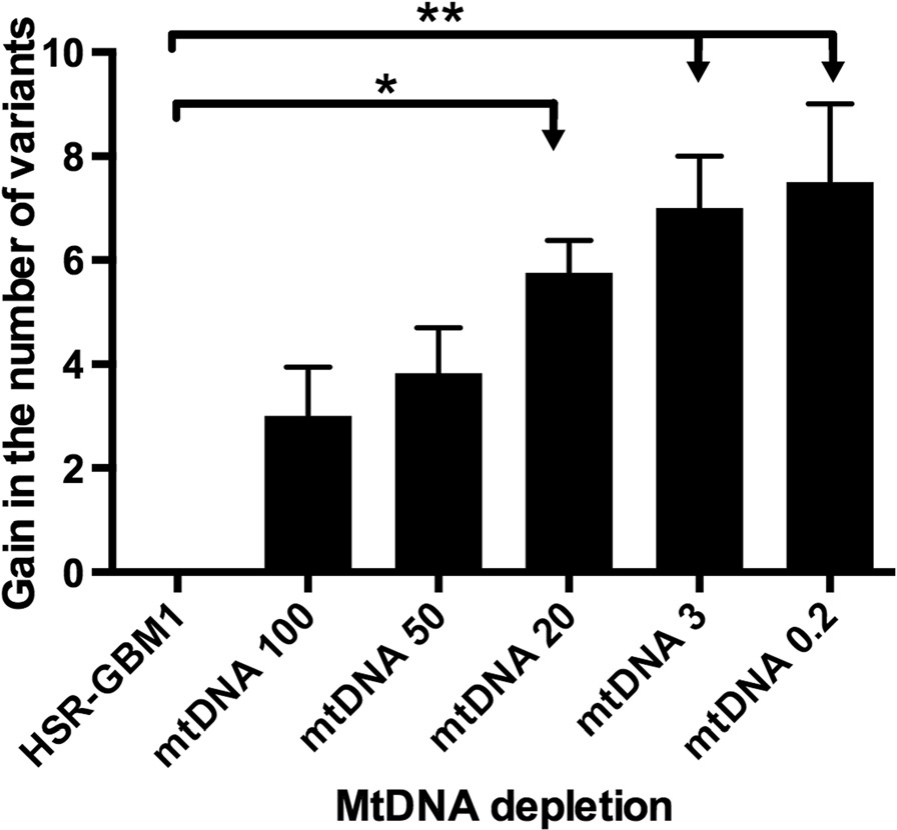
**HSR-GBM1 cells and HSR-GBM1 cells depleted of their mtDNA to levels of 50%, 20%, 3% and 0.2% of their original mtDNA copy number were transplanted into immunedeficient nude mice and developed as tumors with mtDNA copy number being replenished in vivo.** The number of variants gained relative to the non-depleted HSR-GBM1 cells was assessed using HRM. *P = <0.05, **P = <0.01.

Nevertheless, there was an overall gain in the number of variants (Figure 3) as a function of cells having undergone increased levels of mtDNA depletion (P = 0.0027). Specifically, there were significant differences in the incremental gain of variants between the parental cells and the mtDNA^20^ (P < 0.05), mtDNA^3^ (P < 0.01) and mtDNA^0.2^-derived (P < 0.01) tumors (Figure 3). These variants were identified in the other GBM cell lines. The variants that were persistently selected were at 10473 bp (NADH dehydrogenase 4L; ND4L), 10814 bp, 12101 bp and 12102 bp (ND4), 13061 bp (ND5) and 14426 bp (ND6). Interestingly, the variants associated with the ND4 and ND5 were more frequently present in the mtDNA^20^-derived, mtDNA^3^-derived and mtDNA^0.2^-derived tumors, as was the ND6 tumor-specific variant at 14426 bp (Figure 2). We then assessed the presence of variants in cells depleted to 50%, 20% and 3% of their original mtDNA content and then allowed to recover in culture over 14 days. As can be seen from Additional file 5: Table S6, the vast majority of the variants are present in recovered cells, although those variants at 10473 bp (ND4L), 10814 bp, 12101 bp and 12102 bp (ND4) and 13061 bp (ND5) were less frequently present. This suggests that a specific set of mtDNA variants are required to initiate GBM tumorigenesis and that they are selected for whilst those not essential to tumorigenesis appear to be selected against.

### Identification of the tumor-specifying variants in patient samples

In order to determine whether the variants identified in our *in vivo* tumorigenesis model are present in GBM patient samples, we screened high grade tumors obtained from 22 patients diagnosed with glioma (Additional file 5: Table S7). Each of the variants was present in the majority of patient tumor samples with tumor sample T16 sharing all the variants. Furthermore, the variants at 6999 bp (COX I), 8251 bp, 8252 bp (both COX II) and 11361 bp (ND4) were present in all patient tumors whilst the variants at 6999 bp and 11361 bp were also common to all tumors derived from our *in vivo* model.

## Discussion

This is the first study using next generation sequencing to identify the presence of mtDNA variants in GBM cell lines, which are representative of the relapse and/or refractory stage of the disease. We used a two-fold strategy to ensure that the variants identified were present within the cells and were not artifacts. Firstly, we amplified the two overlapping regions of the mitochondrial genome by long PCR followed by next generation sequencing, an approach used by others to, for example, identify the degree of heteroplasmy amongst different populations [29]. Importantly, this strategy ensured that we were not screening and, thus identifying variants, in nuclear pseudo-genes but rather targeting the mitochondrial genome itself [30]. Indeed, isolation of mitochondria and subsequent extraction of mtDNA can result in DNA samples possessing contaminating nuclear DNA. Secondly, we validated the presence of the variants using HRM on genomic DNA, which enabled us to confirm that the variants were ‘true’ calls, and not introduced during long PCR.

Overall, the GBM cell lines harbored proportionally more previously reported variants (16/29) than the normal, unaffected brain samples (20/60) following searches on the MitoMap variant database. Specifically, for the GBM cell lines, 4/13 variants that have not been previously reported resided within the non-coding region and 12/19 within the coding region. For the normal samples, there were two previously unidentified variants in the non-coding region and 18 in the coding region. Indeed, it was most apparent that there were only three variants common to the GBM cell lines and the normal samples, one in the D-loop at position 16519 bp (D-loop) and two in the coding genes at positions 14159 bp and 14160 bp (*ND6*). The 16519 bp variant has been identified in multiple studies [31] and was present in both cohorts at similar levels. However, the 14159 bp and 14160 bp variants have not been previously reported. Consequently, it appears that two distinct populations of variants define the two cohorts.

Although the variants identified in the GBM and normal cohorts appear to be randomly distributed throughout the mitochondrial genome, certain regions were not affected. No variants were identified in the NADH dehydrogenase 1 *(ND1)* gene or the 22 tRNAs suggesting that the incorporation of variants was highly selective to the other 12 coding genes and the D-loop region. Moreover, different regions of the mitochondrial genome were more susceptible to mutation in the normal samples than the GBM cell lines. For the GBM cells, *ND6* and *ND4* were the most susceptible genes in the coding region whilst the origin of light strand replication was the most susceptible non-coding gene. The *ND6*, *ND4* and D-loop variants were also present in the majority of patient samples. For the normal samples, the D-loop was the most susceptible non-coding region whilst *CYT B* was the most susceptible gene in the coding region. Interestingly, for both cohorts, *COX I* was the least susceptible. COX I is one of the subunits of complex IV of the electron transfer chain protein and is amongst the most conserved of the proteins encoded by mtDNA [32]. Nevertheless, the *COX II* and Cytochrome C Oxidase III *(COX III)* genes, which also encode complex IV subunits, were more frequently susceptible to mutation than *COX I* in the GBM cell lines (2.26 fold) and the normal samples (7.87 fold), respectively. CYT B is also highly conserved [32] and the presence of variants in this gene would explain why they were predicted to be disease causing.

Although there was overlap between the various regions of the mitochondrial genome that were affected, there were regions that were specific to each cohort. The variants detected in the origin of light strand replication, *COX II* and *ND4L* were specific to the GBM cell lines, whereas variants specific to the normal samples were detected in the NADH dehydrogenase 2 *(ND2)*, NADH dehydrogenase 3 *(ND3)*, ATPase 6 *(ATP6)*, ATPase 8 *(ATP8)* and *COX III* genes. This suggests that tumorigenesis targets specific regions of the mitochondrial genome for mutagenesis. To ascertain whether our outcomes were not biased by the cell lines and samples used, we confirmed through PHYLIP analysis that the cell lines and normal samples were from similar lineages and that their respective variants were not indicative of clustered cohorts. Furthermore, as many of the variants have not been previously reported, it appears that we have identified a series of mtDNA variants that are indicative of GBM, which can be validated in a large cohort.

Mitochondrial genetics is becoming increasingly important to our understanding of aging, metabolic disease, demographic patterns of human migration and cancer [33]. However, there is no association between mitochondrial dysfunction and the development of tumors in patients diagnosed with mitochondrial disease, where a single variant or deletion is present at high levels [34]. Moreover, no association has been effectively described for the existence of specific variants with a potential hotspot region for mutation [33]. Furthermore, others have argued that the nuclear genome is primarily responsible for the onset of tumorigenesis [35,36]. However, multiple mtDNA variants scattered throughout the mitochondrial genome [37,38] and in specific genes, such as *ND5*, are associated with the progression of tumorigenesis [39]. Nevertheless, studies using mouse transmitochondrial cybrids, where specific mtDNA variants have been introduced into cells with identical nuclear backgrounds, have shown that G→A at position 13997 bp and a frame-shift mutation at position 13885 bp caused by the insertion of a C residue, in the *ND6* gene, were involved in metastasis and can affect tumor phenotype [40]. Other cybrid studies have also shown that mtDNA mutations act to positively induce tumorigenesis [41-45]. However, others hypothesise that there is active selection against unfavourable mtDNA mutations in tumor cells, due to the monoclonal origin of tumors whilst normal tissues are highly polyclonal in nature, thus promoting the selection of non-mutated mtDNA [46].

There are a number of studies that do not support the presence of mitochondrial mutations as a cause for tumorigenesis. In 2001, Kirches et al. analysed the D-loop of 17 paired GBM samples for mtDNA mutations, only to observe that 7 of the 17 tumors possessed variants [47]. The same authors later published a short report describing the absence of any sequence variant in 10 paired astrocytoma samples [48]. Moreover, in a study that focused on mtDNA mutations in mouse brain tumors, no pathogenic variants were detected across the mitochondrial genome in both induced and spontaneously developed tumors [49]. Seoane et al. also argued that the maintenance of mtDNA integrity is essential in order for tumor cells to survive increased reactive oxygen species production, which is associated with tumorigenesis [50,51]. Altogether, it remains controversial whether mtDNA mutations arise as a cause or consequence of tumorigenesis. However, our data support the role of mtDNA as an initiator of tumorigenesis.

From our results, not only were the vast majority of HSR-GBM1 specific variants reestablished in cells depleted to varying degrees, but new variants were introduced as a function of mtDNA depletion. This is highlighted by those tumors generated from cells depleted to 20%, 3% and 0.2% of their original copy number. Interestingly, those variants that had been identified in the MitoMap database were present in all or nearly all of the newly formed tumors suggesting that there is a common core of mtDNA variants in tumors. Furthermore, 5/6 variants that had been established as *de novo* variants in the tumors have not been previously reported. To confirm that the newly derived variants were not indicative of clonal expansion from very few residual variants present in the cells, we analysed the raw data from the next generation sequencing runs for the HSR-GBM1 cell line and were able to confirm that these variants had not been called. Consequently, the induction of *de novo* mtDNA variants appears to be essential to the onset of tumorigenesis. Furthermore, our results reaffirm that *ND4* and *ND6* are the most susceptible genes to mutation in GBM and that GBM is likely to be a complex I disorder, which is supported by the one previous finding, prior to next generation sequencing, that complex I along with the D-loop is implicated in GBM [47]. Consequently, complex I would be a pharmaceutical target for the development of potential therapies.

Overall, the repopulation of depleted cells with mitochondria appears to be essential during tumorigenesis, as recently demonstrated [52]. The accumulation of mtDNA variants would result in a dysfunctional electron transfer chain that promotes reduced OXPHOS output, as is the case for GBM [53] and forces cells to adopt glycolysis, supported by low levels of OXPHOS. As a result, mtDNA copy number remains suppressed, which promotes the metabolic state of aerobic glycolysis, first proposed by Otto Warburg in 1956 [9]. This form of metabolism promotes self-renewal and cellular proliferation, which is also favoured by adult and embryonic stem cells [54-57]. However, embryonic and adult stem cells have the potential to differentiate into mature cell types, increase their mtDNA copy number and, consequently, switch from anaerobic metabolism to OXPHOS as their primary method of metabolism [52,56,58,59].

## Conclusion

We have identified a series of novel mtDNA variants by screening GBM cell lines. Mutations at positions 2130 bp within the *16 s rRNA* and 5752 bp within the origin of light strand replication were sites of frequent mutation across the cell lines. Most of the GBM variants identified in the non-coding regions of mtDNA were within the D-loop region, whereas most variants in the coding portion resided within the *ND4* region. However, *ND6* was determined to be the most susceptible mtDNA region for the development of mutation in the GBM cells, given that it contained the most variants following normalization for the different sizes of the mtDNA regions. These outcomes were further endorsed by mtDNA depletion and *in vivo* replenishment during tumorigenesis and analysis of patient tumor samples, which support the hypothesis that mtDNA variants in complex I act as initiators of GBM.

## Competing interests

The authors declare no competing financial interests.

## Author contribution

KYY carried out the collection, assembly, analysis and interpretation of the data, and drafting of the manuscript; AD carried out the collection of data and analysis; JFD carried out the collection and analysis of all the *in vivo* data; GP designed and assisted in the analysis and interpretation of the protein modeling work; SJW carried out the collection of data; DKG carried out data analysis and provided financial support for the study; MM carried out the analysis and interpretation of the data; TGJ designed the *in vivo* work, carried out the analysis and interpretation of the data and provided the GBM cell lines and mice used in this study; JCSJ carried out the analysis and interpretation of the data, designed the experiments, wrote the manuscript and provided financial support. All authors read and approved the final manuscript.

## Supplementary Material

Additional file 1: Table S1 HRM primers used to screen for mtDNA variants. Click here for file

Additional file 2 Supplementary Methods. Click here for file

Additional file 3: Figure S1 Determining the threshold sensitivity of the Ion Torrent PGM with HRM. **(A)** Mutant and wild-type recombinant plasmids containing a 223 bp amplicon carrying the MELAS A3243G mutation were sequenced using capillary sequencing. **(B)** Sensitivity levels of the Ion Torrent to detect specific percentage of mutation generated by mixing wild-type and mutant plasmid DNA. Confirmation of variant detection is also indicated for HRM. **(C)** The mutant/wild-type specific dilutions at various known percentages were examined by HRM and the results from the difference curves obtained are presented. The 100% mutant plasmid DNA is set as the baseline for comparison for this experiment. Click here for file

Additional file 4: Figure S2 Example of the HRM analysis conducted to confirm the presence of mtDNA variants on the GBM cell lines HK301, SF-767 and BAH1. Click here for file

Additional file 5: Table S2 Normalisation of the identified variants to the size of the mtDNA region on which they reside to determine susceptibility of each region to the acquisition of mutations, based on the variant screen across all GBM cell lines. **Table S3.** Comparison of the different mtDNA regions to their susceptibility to develop mutations based on normalised data against the size of the different mtDNA regions. **Table S4.** Susceptibility table for each mtDNA region, based on the next generation sequencing results obtained from normal brain samples, to the development of variants. The size of each mtDNA region was normalised to allow for comparative analyses to identify the regions most susceptible and least susceptible to mutation. **Table S5.** Fold change comparison of the different susceptibility scores calculated for each mtDNA region of normal brain samples based on the information from Additional file 5: Table S4. **Table S6.** HRM analysis of HSR-GBM1 cells depleted to 50% (mtDNA^50^), 20% (mtDNA^20^) and 3% (mtDNA^3^) of their original mtDNA content. They were then allowed to recover in culture over 14 days and screened by HRM using the panel of primers for the variants identified from the GBM cell lines in Tables 1 and 2, and Figure 2 to determine whether they harbored the same mtDNA variants. **Table S7.** HRM analysis on 22 GBM patient tumor samples screened using the panel of primers for the variants identified from the GBM cell lines in Tables 1, 2 and Figure 2 to identify whether these tumor samples harbor the same mtDNA variants. Click here for file

Additional file 6: Figure S3 Protein modeling of bovine complex IV of the mitochondrial respiratory chain, with identification of the COX I V366M mutation. **(A)** The catalytic centre of complex IV is represented in its entirety with subunits COX I (purple), II (blue) and III (green). Several of the key components are shown; heme *a* and *a*_*3*_ reside within COX I. Heme *a*_*3*_ and the copper B (Cu_B_) centre together form a bimetallic site. Through these redox cofactors an electron transfer process occurs, which is initiated from ferrocytochrome c transferring electrons to the copper A (Cu_A_) site. The cascade of electron flow occurs as follows from the Cu_A_ centre: heme *a* to the heme *a*_*3*_*-* Cu_B_ site, which binds to and reduces molecular oxygen to water. The magnesium (Mg^2+^) centre functions to maintain activity of complex IV. **(B)** The valine to methionine substitution (yellow) is shown at position 366 of the COX I protein subunit, residing within a transmembrane helical domain close to COX II and the intermembrane space. **(C)** Magnified view of the V366M mutation. Click here for file

Additional file 7: Figure S4 Location of the proline to threonine (P223T) mutation in the bovine COX II protein subunit. **(A)** The P223T mutation resides within a helical segment of COX II in the mitochondrial intermembrane space. **(B)** Magnified observation of the same P223T mutation. Click here for file

Additional file 8: Figure S5 Phylogenetic analysis using the PHYLIP software package to determine the evolutionary relationships between the GBM cell lines and normal brain samples. Click here for file
