# Supplementary material for: The identification of mitochondrial DNA variants in glioblastoma multiforme
Source: Acta Neuropathol Commun. 2014 Jan 2;2:1. doi: 10.1186/2051-5960-2-1 (PMC3912901; doi:10.1186/2051-5960-2-1)
Supplement: Additional file 1: Table S1 — HRM primers used to screen for mtDNA variants. [file 2051-5960-2-1-S1.DOCX]

**Additional file 1: Table S1: HRM primers used to screen for mtDNA variants**

| Region on mtDNA | Variant positions | Forward primer (5’→3’) | Reverse primer (5’→3’) | Product size (bp) |
| --- | --- | --- | --- | --- |
| D loop | 194 | TGATTCCTGCCTCATCCTATT | AAGTGGCTGTGCAGACATTC | 131 |
| D loop | 302, 310 | CAGGCGAACATACTTACTAAAG | TTAGGCTGGTGTTAGGGTTC | 184 |
| D loop | 16186, 16218 | CCACCATGAATATTGTACGGTA | TGGCTTTGGAGTTGCAGTTG | 147 |
| 12S rRNA | 1386 | CAAGGTGTAGCCCATGAGGT | CTGTTCAACTAAGCACTCTAC | 132 |
| 16S rRNA | 2130 | CACAGAACCCTCTAAATCCC | ACGCTTTCTTAATTGGTGGCTG | 145 |
| Origin of light strand replication | 5752 | CAGCTAAGCACCCTAATCAAC | TCTAAAGACAGGGGTTAGGC | 161 |
| COX I | 6422 | CTTCTCCTTACACCTAGCAG | GTAGGACTGCTGTGATTAGG | 123 |
| COX I | 6999 | CTCTGAGCCCTAGGATTCAT | CAGTGAATGAAGCCTCCTATG | 152 |
| COX II | 8251, 8252 | CTCTGAAATCTGTGGAGCAAAC | CTAAGTTAGCTTTACAGTGGG | 131 |
| ND4L | 10473 | GGATTAGACTGAACCGAATTG | AGTAGGGAGGATATGAGGTG | 175 |
| ND4 | 10814 | CCTAAACCTACTCCAATGCT | AGGAAAAGGTTGGGGAACAG | 186 |
| ND4 | 11361 | GGCTCACTAAACATTCTACTAC | CTGCGGCAAGTACTATTGAC | 165 |
| ND4 | 11512 | CGCTGGGTCAATAGTACTTG | GTTATAATTATGCCTCATAGGGATAG | 140 |
| ND4 | 11674 | CTGCCTACGACAAACAGAC | GGCAGAATAGTAATGAGGATG | 160 |
| ND4 | 12101, 12102 | CTCACTCACCCACCACATTA | CAGTTCTTGTGAGCTTTCTC | 216 |
| ND5 | 13061 | CAAATCAGCCCAATTAGGTC | GGATTAGTGGGCTATTTTCTGCT | 154 |
| ND6 | 14159, 14160 | TCTTCCCACTCATCCTAACC | TTAGTAGTAGTTACTGGTTGAAC | 109 |
| ND6 | 14426 | TCATACTCTTTCACCCACAG | GGTTGTCTTTGGATATACTACAG | 137 |
| CYTB | 15264, 15267 | GAGGCCAAATATCATTCTGAG | GTTGTTTGATCCCGTTTCGTG | 218 |
