# Supplementary material for: The identification of mitochondrial DNA variants in glioblastoma multiforme
Source: Acta Neuropathol Commun. 2014 Jan 2;2:1. doi: 10.1186/2051-5960-2-1 (PMC3912901; doi:10.1186/2051-5960-2-1)
Supplement: Additional file 2 — Supplementary Methods. [file 2051-5960-2-1-S2.docx]

**Additional file 2: Supplementary Methods**

To determine the efficiency and reproducibility of the Ion Torrent PGM to detect mtDNA variants, we inserted a 223 bp mtDNA amplicon, containing either a known mutation (A3243G) or the wild-type (WT) amplicon, into the pCR2.1 plasmid. WT and mutant plasmids were mixed at specific ratios (mutant:WT – 0:100; 1:99; 3:97; 20:80; 100:0) for detection (Additional file 2: Figure S1A). The mutant molecule was detected at or close to its prescribed ratio by Ion Torrent following the use of rigorous inclusion and exclusion criteria (Fig S1B).

The same samples were then re-analysed, along with additional ratios of WT and mutant DNA, by HRM (F: 5’-AGCGCCTTCCCCCGTAAATG-3’, R: 5’-TTCGTTCGGTAAGCATTAGG-3’) to determine whether HRM variant calling was representative of Ion Torrent base calling and could be used to validate Ion Torrent outputs (Additional file 2: Figure S1C). With the 100% WT plasmid sample set as a baseline for analysis, we observed almost identical melt profiles for samples containing the same ratios of mutant and wild type plasmids with the 3% mutant molecule being clearly identifiable (Additional file 2: Figure S1C).

Based on the combined outcomes of the Ion Torrent and HRM analyses, we set in place a two-fold screening approach. We set a 3% threshold for variant detection on the Ion Torrent, since the presence of variants below this value is likely to arise from background noise or false positives. We then confirmed the presence or absence of these variants using HRM analysis. Primers are listed in Additional file 1: Table S1.
