# Supplementary figures and images for: The identification of mitochondrial DNA variants in glioblastoma multiforme
Source: Acta Neuropathol Commun. 2014 Jan 2;2:1. doi: 10.1186/2051-5960-2-1 (PMC3912901; doi:10.1186/2051-5960-2-1)

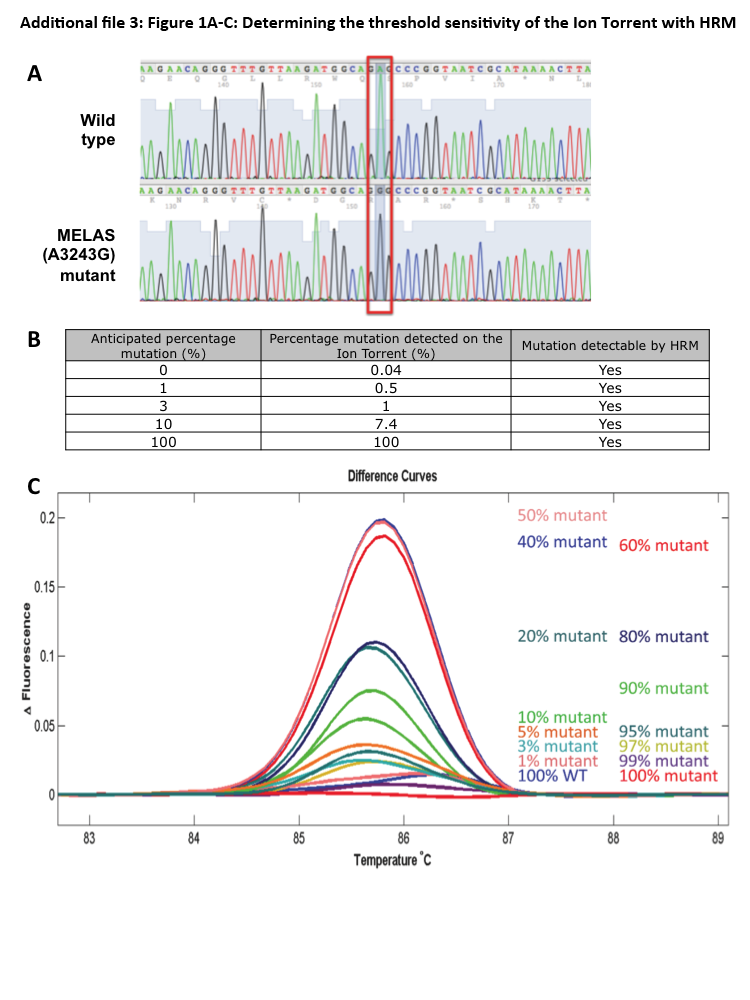

Supplement: Additional file 3: Figure S1 — Determining the threshold sensitivity of the Ion Torrent PGM with HRM. (A) Mutant and wild-type recombinant plasmids containing a 223 bp amplicon carrying the MELAS A3243G mutation were sequenced using capillary sequencing. (B) Sensitivity levels of the Ion Torrent to detect specific percentage of mutation generated by mixing wild-type and mutant plasmid DNA. Confirmation of variant detection is also indicated for HRM. (C) The mutant/wild-type specific dilutions at various known percentages were examined by HRM and the results from the difference curves obtained are presented. The 100% mutant plasmid DNA is set as the baseline for comparison for this experiment. [file 2051-5960-2-1-S3.TIFF]

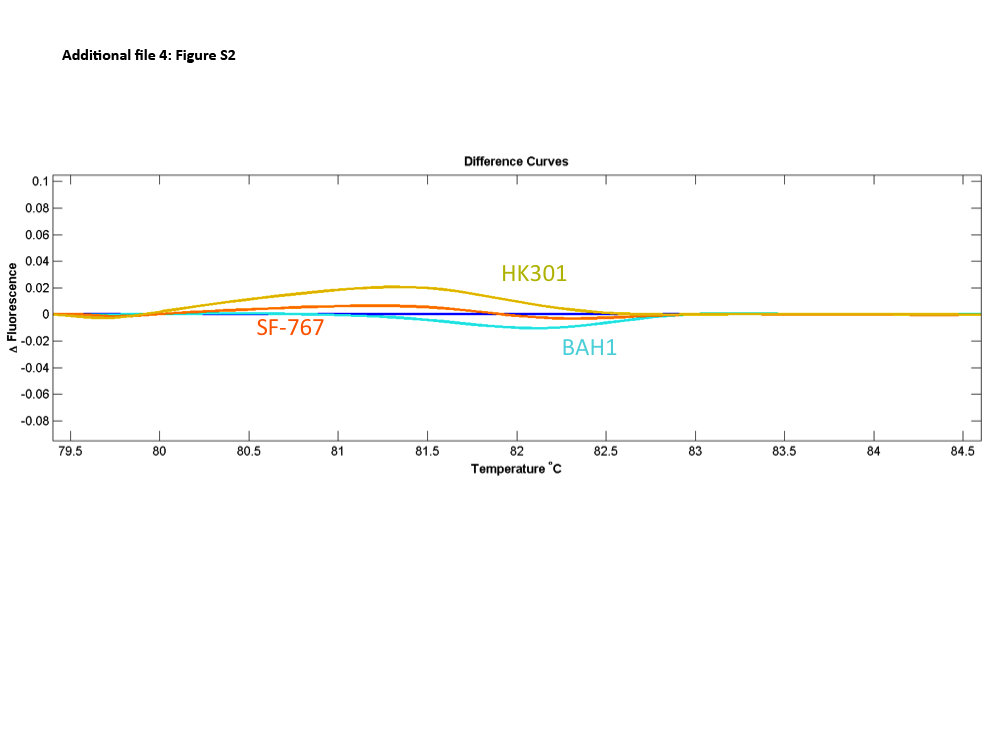

Supplement: Additional file 4: Figure S2 — Example of the HRM analysis conducted to confirm the presence of mtDNA variants on the GBM cell lines HK301, SF-767 and BAH1. [file 2051-5960-2-1-S4.TIFF]

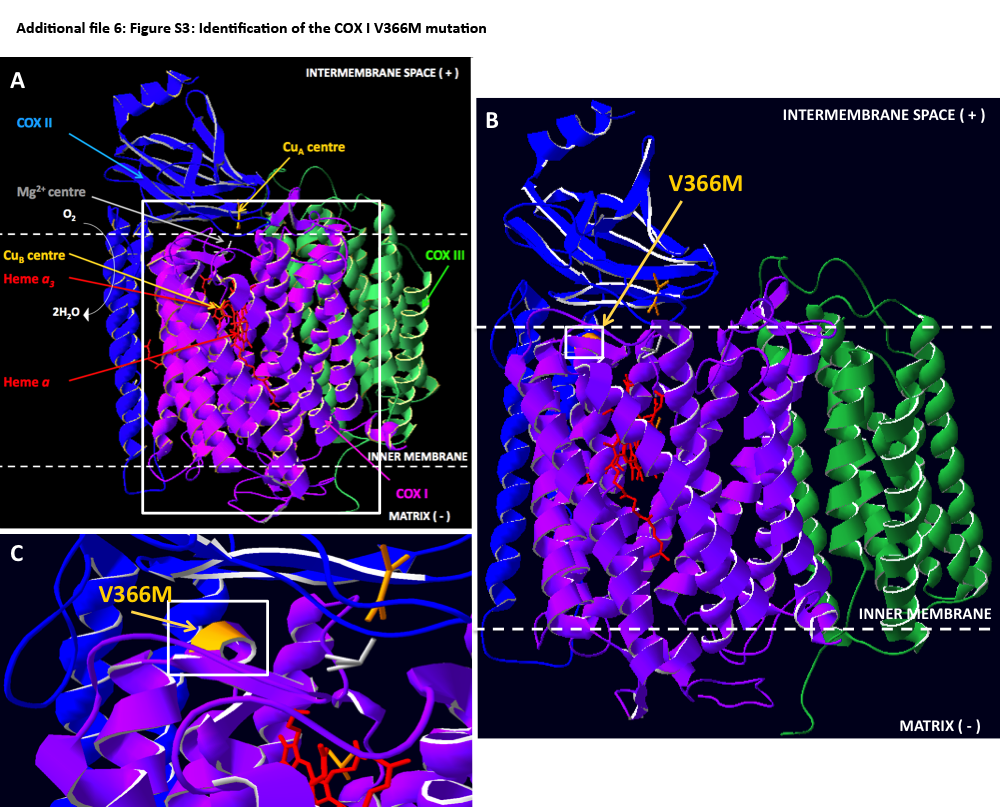

Supplement: Additional file 6: Figure S3 — Protein modeling of bovine complex IV of the mitochondrial respiratory chain, with identification of the COX I V366M mutation. (A) The catalytic centre of complex IV is represented in its entirety with subunits COX I (purple), II (blue) and III (green). Several of the key components are shown; heme a and a 3 reside within COX I. Heme a 3 and the copper B (CuB) centre together form a bimetallic site. Through these redox cofactors an electron transfer process occurs, which is initiated from ferrocytochrome c transferring electrons to the copper A (CuA) site. The cascade of electron flow occurs as follows from the CuA centre: heme a to the heme a 3 - CuB site, which binds to and reduces molecular oxygen to water. The magnesium (Mg2+) centre functions to maintain activity of complex IV. (B) The valine to methionine substitution (yellow) is shown at position 366 of the COX I protein subunit, residing within a transmembrane helical domain close to COX II and the intermembrane space. (C) Magnified view of the V366M mutation. [file 2051-5960-2-1-S6.TIFF]

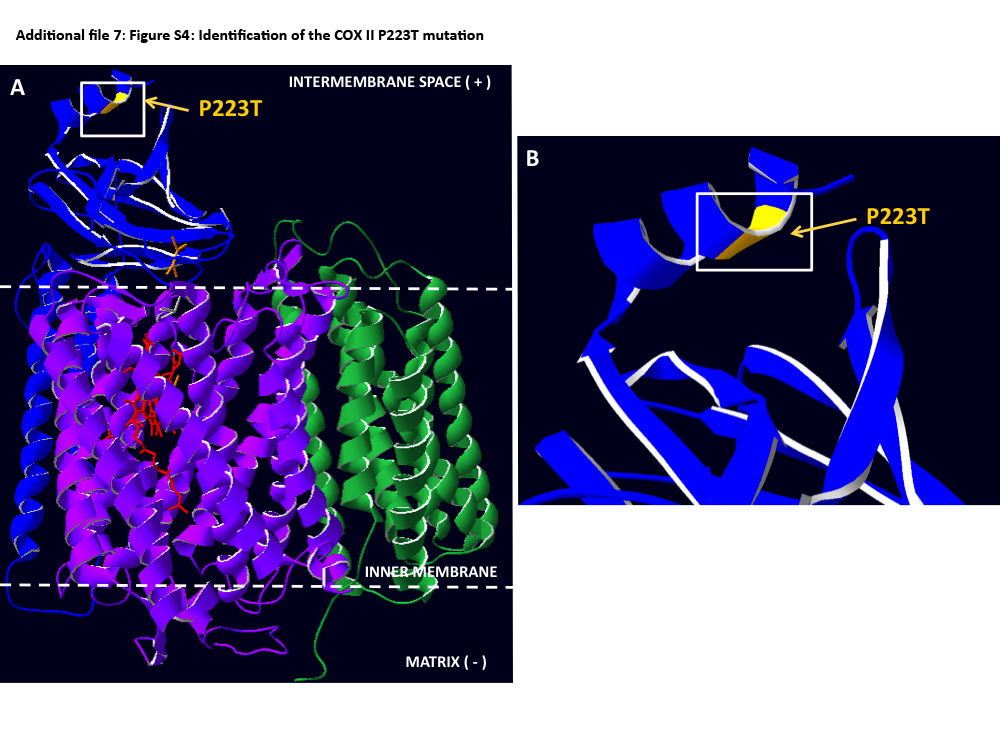

Supplement: Additional file 7: Figure S4 — Location of the proline to threonine (P223T) mutation in the bovine COX II protein subunit. (A) The P223T mutation resides within a helical segment of COX II in the mitochondrial intermembrane space. (B) Magnified observation of the same P223T mutation. [file 2051-5960-2-1-S7.TIFF]

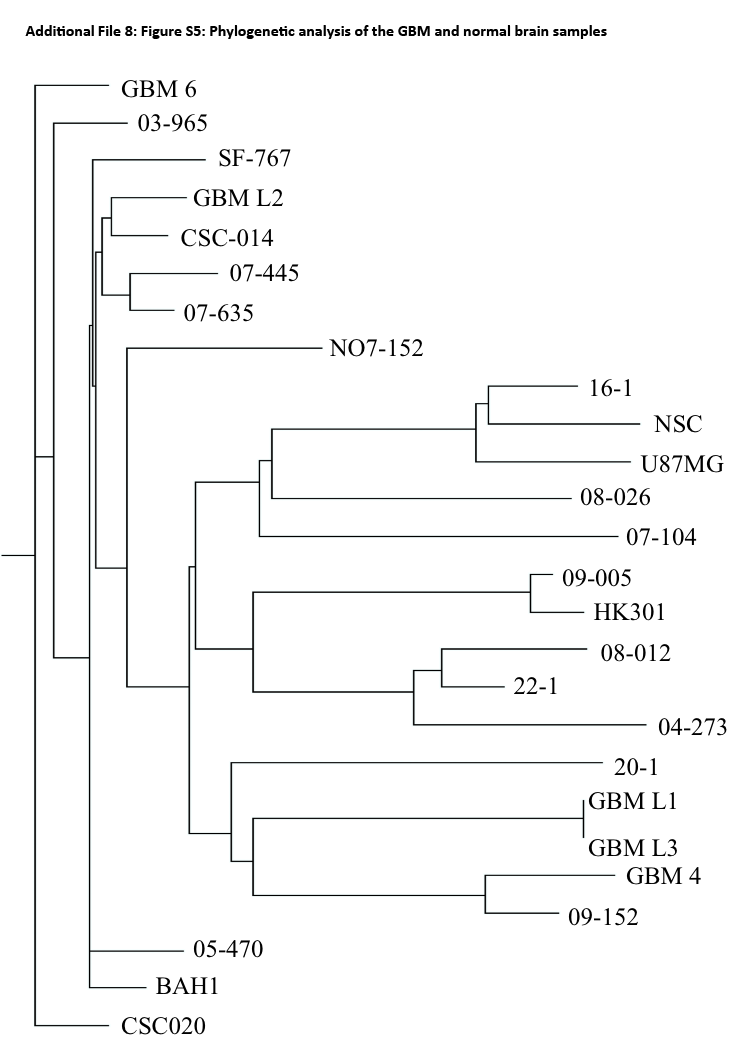

Supplement: Additional file 8: Figure S5 — Phylogenetic analysis using the PHYLIP software package to determine the evolutionary relationships between the GBM cell lines and normal brain samples. [file 2051-5960-2-1-S8.TIFF]
