# Supplementary material for: The identification of mitochondrial DNA variants in glioblastoma multiforme
Source: Acta Neuropathol Commun. 2014 Jan 2;2:1. doi: 10.1186/2051-5960-2-1 (PMC3912901; doi:10.1186/2051-5960-2-1)
Supplement: Additional file 5: Table S2 — Normalisation of the identified variants to the size of the mtDNA region on which they reside to determine susceptibility of each region to the acquisition of mutations, based on the variant screen across all GBM cell lines. Table S3. Comparison of the different mtDNA regions to their susceptibility to develop mutations based on normalised data against the size of the different mtDNA regions. Table S4. Susceptibility table for each mtDNA region, based on the next generation sequencing results obtained from normal brain samples, to the development of variants. The size of each mtDNA region was normalised to allow for comparative analyses to identify the regions most susceptible and least susceptible to mutation. Table S5. Fold change comparison of the different susceptibility scores calculated for each mtDNA region of normal brain samples based on the information from Additional file 5: Table S4. Table S6. HRM analysis of HSR-GBM1 cells depleted to 50% (mtDNA50), 20% (mtDNA20) and 3% (mtDNA3) of their original mtDNA content. They were then allowed to recover in culture over 14 days and screened by HRM using the panel of primers for the variants identified from the GBM cell lines in Tables 1 and 2, and Figure 2 to determine whether they harbored the same mtDNA variants. Table S7. HRM analysis on 22 GBM patient tumor samples screened using the panel of primers for the variants identified from the GBM cell lines in Tables 1, 2 and Figure 2 to identify whether these tumor samples harbor the same mtDNA variants. [file 2051-5960-2-1-S5.DOCX]

**Additional file 5: Table S2: Normalisation of identified variants to the size of the mtDNA genome**

|  |  |  | | |  | |  | |  | Normalised data | |
| --- | --- | --- | --- | --- | --- | --- | --- | --- | --- | --- | --- |
| Reference Position | Gene region | | | Nucleotide positions for gene regions (bp) | | Size of gene region (bp) | | Number of variants detected in gene region | | Probability of mutation in each gene region | Probability of mutation grouped for coding/non-coding regions |
| 16186, 16218,16224, 16519...194, 302, 310 | Non-coding region | | D loop | 1..576, 16024..16569 | | 1121 | | 7 | | 0.006244425 | 0.002083000 |
| 1386 |  |  | 12s rRNA | 648..1601 | | 953 | | 1 | | 0.001049318 |  |
| 2002, 2130, 2817, 3168 |  |  | 16s rRNA | 1671..3229 | | 1558 | | 4 | | 0.002567394 |  |
| 5752 |  |  | L strand replication origin | 5721..5798 | | 77 | | 1 | | 0.012987013 |  |
| 6422, 6999 | Coding region | | Cytochrome C Oxidase I | 5904..7445 | | 1541 | | 2 | | 0.001297859 | 0.001839659 |
| 8251, 8252 |  |  | Cytochrome C Oxidase II | 7586..8269 | | 683 | | 2 | | 0.002928258 |  |
| 10473 |  |  | NADH dehydrogenase 4L | 10470..10766 | | 296 | | 1 | | 0.003378378 |  |
| 10814, 11361, 11512, 11674, 12101, 12102 |  |  | NADH dehydrogenase 4 | 10760..12137 | | 1377 | | 6 | | 0.004357298 |  |
| 12877, 13043, 13061 |  |  | NADH dehydrogenase 5 | 12337..14148 | | 1811 | | 3 | | 0.001656543 |  |
| 14159, 14160, 14426 |  |  | NADH dehydrogenase 6 | 14149..14673 | | 524 | | 3 | | 0.005725191 |  |
| 15264, 15267 |  |  | Cytochrome B | 14747..15887 | | 1140 | | 2 | | 0.001754386 |  |

**Additional file 5: Table S3: Comparison of the different mtDNA regions to their susceptibility to develop mutations**

| Gene region | D loop | 12s rRNA | 16s rRNA | L strand origin | COX I | COX II | ND4L | ND4 | ND5 | ND6 | CYT B |
| --- | --- | --- | --- | --- | --- | --- | --- | --- | --- | --- | --- |
| D loop |  | 5.95 | 2.43 | 0.48 | 4.81 | 2.13 | 1.85 | 1.43 | 3.77 | 1.09 | 3.56 |
| 12s rRNA | 0.17 |  | 0.41 | 0.08 | 0.81 | 0.36 | 0.31 | 0.24 | 0.63 | 0.18 | 0.60 |
| 16s rRNA | 0.41 | 2.45 |  | 0.20 | 1.98 | 0.88 | 0.76 | 0.59 | 1.55 | 0.45 | 1.46 |
| L strand origin | 2.08 | 12.38 | 5.06 |  | 10.01 | 4.44 | 3.84 | 2.98 | 7.84 | 2.27 | 7.40 |
| COX I | 0.21 | 1.24 | 0.51 | 0.10 |  | 0.44 | 0.38 | 0.30 | 0.78 | 0.23 | 0.74 |
| COX II | 0.47 | 2.79 | 1.14 | 0.23 | 2.26 |  | 0.87 | 0.67 | 1.77 | 0.51 | 1.67 |
| ND4L | 0.54 | 3.22 | 1.32 | 0.26 | 2.60 | 1.15 |  | 0.78 | 2.04 | 0.59 | 1.93 |
| ND4 | 0.70 | 4.15 | 1.70 | 0.34 | 3.36 | 1.49 | 1.29 |  | 2.63 | 0.76 | 2.48 |
| ND5 | 0.27 | 1.58 | 0.65 | 0.13 | 1.28 | 0.57 | 0.49 | 0.38 |  | 0.29 | 0.94 |
| ND6 | 0.92 | 5.46 | 2.23 | 0.44 | 4.41 | 1.96 | 1.69 | 1.31 | 3.46 |  | 3.26 |
| CYT B | 0.28 | 1.67 | 0.68 | 0.14 | 1.35 | 0.60 | 0.52 | 0.40 | 1.06 | 0.31 |  |
|  |  |  |  |  |  |  |  |  |  |  |  |
| if value >1 it means that susceptibility of region to variant changes is greater for the gene region listed on the left side than the gene listed across the top | | | | | | | | | | | |
| if value <1 it means that susceptibility of region to variant changes is less for the gene region listed on the left side than the gene listed across the top | | | | | | | | | | | |

**Additional file 5: Table S4: Susceptibility table based on each mtDNA region**

|  |  |  |  |  |  | Normalised data | |
| --- | --- | --- | --- | --- | --- | --- | --- |
| Reference Position | Gene region | | Nucleotide positions for gene regions (bp) | Size of gene region (bp) | Number of variants detected in gene region | Probability of mutation in each gene region | Probability of mutation grouped for coding/non-coding regions |
| 16069, 16126, 16145, 16179, 16222, 16261, 16293, 16356, 16519…9, 10, 66, 73, 150, 189, 195, 242 | Non-coding | D loop | 1..576, 16024..16569 | 1121 | 17 | 0.015165031 | 0.003364845 |
| 751 |  | 12s rRNA | 648..1601 | 953 | 1 | 0.001049318 |  |
| 2232, 2647, 3010 |  | 16s rRNA | 1671..3229 | 1558 | 3 | 0.001925546 |  |
| 4646, 4701, 4703, 4878, 4879 | Coding region | NADH dehydrogenase 2 | 4470..5511 | 1041 | 5 | 0.004803074 | 0.003776143 |
| 6146 |  | Cytochrome C Oxidase I | 5904..7445 | 1541 | 1 | 6.48929E-04 |  |
| 8497 |  | ATPase 8 | 8366..8572 | 206 | 1 | 0.004854369 |  |
| 8630, 8756, 8790, 8994, 9070 |  | ATPase 6 | 8527..9207 | 680 | 5 | 0.007352941 |  |
| 9258, 9526, 9528, 9558 |  | Cytochrome C Oxidase III | 9207..9990 | 783 | 4 | 0.005108557 |  |
| 10398 |  | NADH dehydrogenase 3 | 10059..10404 | 345 | 1 | 0.002898551 |  |
| 10993, 11332, 11467, 11516, 11725, 11791 |  | NADH dehydrogenase 4 | 10760..12137 | 1377 | 6 | 0.004357298 |  |
| 12719, 12774, 13984, 13985 |  | NADH dehydrogenase 5 | 12337..14148 | 1811 | 4 | 0.002208724 |  |
| 14155, 14159, 14160 |  | NADH dehydrogenase 6 | 14149..14673 | 524 | 3 | 0.005725191 |  |
| 14770, 14823, 14857, 14861, 14866, 15287, 15452, 15579, 15693 |  | Cytochrome B | 14747..15887 | 1140 | 9 | 0.007894737 |  |

**Additional file 5: Table S5: Fold change comparison of the different susceptibility scores**

| Gene region | D loop | 12s rRNA | 16s rRNA | ND2 | COX I | ATP8 | ATP6 | COX III | ND3 | ND4 | ND5 | ND6 | CYT B |
| --- | --- | --- | --- | --- | --- | --- | --- | --- | --- | --- | --- | --- | --- |
| D loop |  | 14.45 | 7.88 | 3.16 | 23.37 | 3.12 | 2.06 | 2.97 | 5.23 | 3.48 | 6.87 | 2.65 | 1.92 |
| 12s rRNA | 0.07 |  | 0.54 | 0.22 | 1.62 | 0.22 | 0.14 | 0.21 | 0.36 | 0.24 | 0.48 | 0.18 | 0.13 |
| 16s rRNA | 0.13 | 1.84 |  | 0.40 | 2.97 | 0.40 | 0.26 | 0.38 | 0.66 | 0.44 | 0.87 | 0.34 | 0.24 |
| ND2 | 0.32 | 4.58 | 2.49 |  | 7.40 | 0.99 | 0.65 | 0.94 | 1.66 | 1.10 | 2.17 | 0.84 | 0.61 |
| COX I | 0.04 | 0.62 | 0.34 | 0.14 |  | 0.13 | 0.09 | 0.13 | 0.22 | 0.15 | 0.29 | 0.11 | 0.08 |
| ATP8 | 0.32 | 4.63 | 2.52 | 1.01 | 7.48 |  | 0.66 | 0.95 | 1.67 | 1.11 | 2.20 | 0.85 | 0.61 |
| ATP6 | 0.48 | 7.01 | 3.82 | 1.53 | 11.33 | 1.51 |  | 1.44 | 2.54 | 1.69 | 3.33 | 1.28 | 0.93 |
| COX III | 0.34 | 4.87 | 2.65 | 1.06 | 7.87 | 1.05 | 0.69 |  | 1.76 | 1.17 | 2.31 | 0.89 | 0.65 |
| ND3 | 0.19 | 2.76 | 1.51 | 0.60 | 4.47 | 0.60 | 0.39 | 0.57 |  | 0.67 | 1.31 | 0.51 | 0.37 |
| ND4 | 0.29 | 4.15 | 2.26 | 0.91 | 6.71 | 0.90 | 0.59 | 0.85 | 1.50 |  | 1.97 | 0.76 | 0.55 |
| ND5 | 0.15 | 2.10 | 1.15 | 0.46 | 3.40 | 0.45 | 0.30 | 0.43 | 0.76 | 0.51 |  | 0.39 | 0.28 |
| ND6 | 0.38 | 5.46 | 2.97 | 1.19 | 8.82 | 1.18 | 0.78 | 1.12 | 1.98 | 1.31 | 2.59 |  | 0.73 |
| CYT B | 0.52 | 7.52 | 4.10 | 1.64 | 12.17 | 1.63 | 1.07 | 1.55 | 2.72 | 1.81 | 3.57 | 1.38 |  |
|  |  |  |  |  |  |  |  |  |  |  |  |  |  |
| Key |  |  |  |  |  |  |  |  |  |  |  |  |  |
| if value >1 susceptibility of the region to variant changes is greater for the gene listed on the left | | | | | | |  |  |  |  |  |  |  |
| if value <1 susceptibility of the region to variant changes is less for the gene listed on the left | | | | | | |  |  |  |  |  |  |  |

**Additional file 5: Table S6: HRM analysis of HSR-GBM1 depleted cells**

|  | |  | |  | |  | | | |  |  |  |  |  |  |  |
| --- | --- | --- | --- | --- | --- | --- | --- | --- | --- | --- | --- | --- | --- | --- | --- | --- |
|  | | Reference Position | | Variant | | Gene region | | | | mtDNA^50^ - Recovery 14 days | | | mtDNA^20^ - Recovery 14 days | | mtDNA^3^ - Recovery 14 days |  |
| Non-coding region | | 16186 | | C→T | | D-loop | | | |  | | |  | |  |  |
|  |  | 16218 | | C→T | |  |  |  |  |  | | |  | |  |  |
|  |  | 194 | | C→T | |  |  |  |  |  | | |  | |  |  |
|  |  | 302 | | A→C | |  |  |  |  |  | | |  | |  |  |
|  | | 310 | | T→C | |  | | | |  | | |  | |  |  |
|  |  | 1386 | | T→C | | 12s rRNA | | | |  | | |  | |  |  |
|  |  | 2130 | | A→G | | 16s rRNA | | | |  | | |  | |  |  |
|  |  | 5752 | | A→G | | Origin of L-strand replication | | | |  | | |  | |  |  |
| Coding region | | 6422 | | C→T | | COX I | | | |  | | |  | |  |  |
|  |  | 6999 | | G→A | |  |  |  |  |  | | |  | |  |  |
|  |  | 8251 | | G→A | | COX II | | | |  | | |  | |  |  |
|  |  | 8252 | | C→A | |  |  |  |  |  | | |  | |  |  |
|  |  | 10473 | | C→G | | ND4L | | | |  | | |  | |  |  |
|  |  | 10814 | | A→C | | ND4 | | | |  | | |  | |  |  |
|  |  | 11361 | | T→C | |  |  |  |  |  | | |  | |  |  |
|  |  | 11512 | | C→A | |  |  |  |  |  | | |  | |  |  |
|  |  | 11674 | | C→T | |  |  |  |  |  | | |  | |  |  |
|  |  | 12101 | | T→C | |  |  |  |  |  | | |  | |  |  |
|  |  | 12102 | | C→T | |  |  |  |  |  | | |  | |  |  |
|  |  | 13061 | | C→A | | ND5 | | | |  | | |  | |  |  |
|  |  | 14159 | | C→G | | ND6 | | | |  | | |  | |  |  |
|  |  | 14160 | | G→C | |  |  |  |  |  | | |  | |  |  |
|  |  | 14426 | | C→T | |  |  |  |  |  | | |  | |  |  |
|  |  | 15264 | | C→T | | CYT B | | | |  | | |  | |  |  |
|  |  | 15267 | | C→G | |  |  |  |  |  | | |  | |  |  |
|  | |  | |  | |  | | | |  | | |  | |  |  |
|  | Key: | |  | | Variant present | |  |  |  | |  |  |  |  | |  |
|  |  | |  | | Variant absent | |  |  |  | |  |  |  |  | |  |
|  |  | |  | | Variant present - analysed jointly (either 1 present or both) | | | | | | | | | | |  |
|  |  | |  | | Variant absent - analysed jointly (either 1 absent or both) | | | | | | | | | | |  |

**Additional file 5: Table S7: HRM analysis on 22 GBM patient tumor samples**

|  |  |  |  | GBM patient tumor samples | | | | | | | | | | | | | | | | | | | | | |
| --- | --- | --- | --- | --- | --- | --- | --- | --- | --- | --- | --- | --- | --- | --- | --- | --- | --- | --- | --- | --- | --- | --- | --- | --- | --- |
|  | Reference Position | Variant | Gene region | T1 | T2 | T3 | T4 | T5 | T6 | T7 | T8 | T9 | T10 | T11 | T12 | T13 | T14 | T15 | T16 | T17 | T18 | T19 | T20 | T21 | T22 |
| Non-coding region | 16186 | C→T | D-loop |  |  |  |  |  |  |  |  |  |  |  |  |  |  |  |  |  |  |  |  |  |  |
|  | 16218 | C→T |  |  |  |  |  |  |  |  |  |  |  |  |  |  |  |  |  |  |  |  |  |  |  |
|  | 194 | C→T |  |  |  |  |  |  |  |  |  |  |  |  |  |  |  |  |  |  |  |  |  |  |  |
|  | 302 | A→C |  |  |  |  |  |  |  |  |  |  |  |  |  |  |  |  |  |  |  |  |  |  |  |
|  | 310 | T→C |  |  |  |  |  |  |  |  |  |  |  |  |  |  |  |  |  |  |  |  |  |  |  |
|  | 1386 | T→C | 12s rRNA |  |  |  |  |  |  |  |  |  |  |  |  |  |  |  |  |  |  |  |  |  |  |
|  | 2130 | A→G | 16s rRNA |  |  |  |  |  |  |  |  |  |  |  |  |  |  |  |  |  |  |  |  |  |  |
|  | 5752 | A→G | Origin of L-strand replication |  |  |  |  |  |  |  |  |  |  |  |  |  |  |  |  |  |  |  |  |  |  |
| Coding region | 6422 | C→T | COX I |  |  |  |  |  |  |  |  |  |  |  |  |  |  |  |  |  |  |  |  |  |  |
|  | 6999 | G→A |  |  |  |  |  |  |  |  |  |  |  |  |  |  |  |  |  |  |  |  |  |  |  |
|  | 8251 | G→A | COX II |  |  |  |  |  |  |  |  |  |  |  |  |  |  |  |  |  |  |  |  |  |  |
|  | 8252 | C→A |  |  |  |  |  |  |  |  |  |  |  |  |  |  |  |  |  |  |  |  |  |  |  |
|  | 10473 | C→G | ND4L |  |  |  |  |  |  |  |  |  |  |  |  |  |  |  |  |  |  |  |  |  |  |
|  | 10814 | A→C | ND4 |  |  |  |  |  |  |  |  |  |  |  |  |  |  |  |  |  |  |  |  |  |  |
|  | 11361 | T→C |  |  |  |  |  |  |  |  |  |  |  |  |  |  |  |  |  |  |  |  |  |  |  |
|  | 11512 | C→A |  |  |  |  |  |  |  |  |  |  |  |  |  |  |  |  |  |  |  |  |  |  |  |
|  | 11674 | C→T |  |  |  |  |  |  |  |  |  |  |  |  |  |  |  |  |  |  |  |  |  |  |  |
|  | 12101 | T→C |  |  |  |  |  |  |  |  |  |  |  |  |  |  |  |  |  |  |  |  |  |  |  |
|  | 12102 | C→T |  |  |  |  |  |  |  |  |  |  |  |  |  |  |  |  |  |  |  |  |  |  |  |
|  | 13061 | C→A | ND5 |  |  |  |  |  |  |  |  |  |  |  |  |  |  |  |  |  |  |  |  |  |  |
|  | 14159 | C→G | ND6 |  |  |  |  |  |  |  |  |  |  |  |  |  |  |  |  |  |  |  |  |  |  |
|  | 14160 | G→C |  |  |  |  |  |  |  |  |  |  |  |  |  |  |  |  |  |  |  |  |  |  |  |
|  | 14426 | C→T |  |  |  |  |  |  |  |  |  |  |  |  |  |  |  |  |  |  |  |  |  |  |  |
|  | 15264 | C→T | CYT B |  |  |  |  |  |  |  |  |  |  |  |  |  |  |  |  |  |  |  |  |  |  |
|  | 15267 | C→G |  |  |  |  |  |  |  |  |  |  |  |  |  |  |  |  |  |  |  |  |  |  |  |
|  |  |  |  |  |  |  |  |  |  |  |  |  |  |  |  |  |  |  |  |  |  |  |  |  |  |
|  | Key: |  | Variant present |  |  |  |  |  |  |  |  |  |  |  |  |  |  |  |  |  |  |  |  |  |  |
|  |  |  | Variant absent |  |  |  |  |  |  |  |  |  |  |  |  |  |  |  |  |  |  |  |  |  |  |
|  |  |  | Variant present - analysed jointly (either 1 present or both) | | | | | | | |  |  |  |  |  |  |  |  |  |  |  |  |  |  |  |
|  |  |  | Variant absent - analysed jointly (either 1 absent or both) | | | | | | | |  |  |  |  |  |  |  |  |  |  |  |  |  |  |  |
